# Dynamic monitoring of a core pro-inflammatory cytokine panel improves mortality prediction in severe influenza

**DOI:** 10.3389/fimmu.2026.1805650

**Published:** 2026-08-19

**Authors:** Kailin Mai, Yang Wang, Wei Qu, Yunceng Weng, Zhengshi Lin, Liping Chen, Arlindo Oliveira, Nanshan Zhong, Chitin Hon, Weiqi Pan, Zifeng Yang

**Affiliations:** 1 Henan University College of Medicine, Kaifeng, China; 2 School of Life Sciences, Henan University, Kaifeng, China; 3 National Clinical Research Center for Respiratory Disease, State Key Laboratory of Respiratory Disease, Guangzhou Institute of Respiratory Health, The First Affiliated Hospital of Guangzhou Medical University, Guangzhou, China; 4 China-Portugal Belt and Road Joint Laboratory on AI and Public Health Issues, the First Affiliated Hospital of Guangzhou Medical University, Guangzhou Medical University, Guangzhou, China; 5 Hengqin Hospital, The First Affiliated Hospital of Guangzhou Medical University, Hengqin, China; 6 Respiratory Disease AI Laboratory on Epidemic Intelligence and Medical Big Data Instrument Applications, Faculty of Innovative Engineering, Macau University of Science and Technology, Macau, China; 7 College of Sciences, China Jiliang University, Hangzhou, China; 8 INESC-ID, Instituto Superior Técnico, Universidade de Lisboa, Lisbon, Portugal; 9 Guangzhou National Laboratory, Guangzhou, China; 10 Department of Engineering Science, Faculty of Innovation Engineering, Macau University of Science and Technology, Macau, China

**Keywords:** biomarkers, cytokines, longitudinal analysis, risk stratification, severe influenza

## Abstract

**Background:**

Hypercytokinemia is a major contributor of tissue damage and mortality in severe influenza. However, most studies rely on static, single-time-point measurements, providing limited insight into the evolving inflammatory response. The prognostic relevance of longitudinal cytokine trajectories remains poorly defined.

**Methods:**

We conducted a multi-cohort analysis of 186 patients from three public datasets to identify cytokines with influenza-severity-dependent expression patterns. We then analyzed a longitudinal cohort of 33 patients with severe influenza who underwent serial cytokine measurements, using linear mixed-effects models to identify the top 10 severity-associated cytokines. Integrating both analyses, we defined a core cytokine panel and a composite core panel score. Prognostic value was assessed using time-varying Cox regression and landmark analyses, with comparisons against clinical predictors, including LODS scores and CRP, as well as sensitivity analyses adjusting for diabetes.

**Results:**

A core panel of six pro-inflammatory cytokines (IL-8, IL-6, G-CSF, MCP-1, TNF-α, and MIP-1α) was identified. Non-survivors showed distinct longitudinal trajectories of the core panel score, characterized by a progressive increase over the disease course. Paired sputum analyses suggested that these systemic cytokine signatures partially reflected pulmonary inflammation. Baseline core panel scores were not significantly associated with mortality (HR 3.29, 95% CI 0.95-11.38; *P* = 0.059), whereas time-varying monitoring showed better model fit (AIC 29.34 vs 34.85). Core panel scores remained associated with mortality in analyses accounting for LODS scores, baseline CRP, and diabetes. Landmark analysis further suggested that updated core panel scores may provide increasing prognostic information over time compared with baseline assessment, with delta AUC increasing from near zero at day 5 to 0.24 by day 17 after ICU admission.

**Conclusions:**

Dynamic monitoring of a core cytokine panel may provide greater prognostic information than single-time-point assessment in severe influenza, although these findings require validation in larger independent cohorts. Longitudinal inflammatory profiling may help refine risk stratification and provide a framework for future precision studies.

## 1 Introduction

Influenza remains a major global public health threat, causing substantial morbidity and mortality worldwide. Despite the widespread implementation of vaccination programs and the availability of antiviral therapies, the World Health Organization estimates that seasonal influenza causes approximately 1 billion infections each year, including 3–5 million cases of severe illness and 290, 000-650, 000 respiratory deaths (1). Notably, severe influenza continues to result in high hospitalization rates and considerable mortality, even among individuals without recognized high-risk comorbidities, underscoring the persistent and unresolved clinical challenge of preventing and managing critical influenza illness (2).

Severe influenza often progresses to acute respiratory distress syndrome (ARDS), sepsis, and multi-organ failure, with mortality rates of approximately 20% in ICU patients (2, 3). Although antiviral therapies effectively reduce viral load, their ability to improve survival remains limited once critical illness has developed. In our previous reports on patients with highly pathogenic avian influenza infection, potent antiviral treatment rapidly suppressed viral replication and pulmonary inflammation but failed to substantially attenuate systemic cytokine elevations, which were markedly higher in non-survivors (4). These findings support the concept that dysregulated host immune responses, particularly hypercytokinemia, play a central role in driving tissue damage and organ dysfunction (5). Consequently, immunomodulatory interventions have attracted increasing attention as potential adjunctive therapies (6). However, clinical trials evaluating these approaches have yielded inconsistent results (7), highlighting persistent uncertainty regarding patient selection and the optimal timing of immune-targeted therapies.

A critical limitation of current biomarker studies in severe influenza is their reliance on static, single-time-point measurements of inflammatory mediators (8), which fail to capture the dynamic evolution of the host immune response. Moreover, reported cytokine signatures often vary across cohorts, reflecting substantial heterogeneity among patients with severe influenza. As a result, the prognostic value of longitudinal cytokine trajectories for tracking disease progression, stratifying mortality risk, and identifying optimal windows for immunomodulatory intervention remains insufficiently explored. This knowledge gap has hindered the translation of inflammatory biomarkers into precision therapeutic strategies.

In this study, we first integrated publicly available cytokine datasets to identify candidate cytokines with influenza-severity-dependent expression patterns. We then combined these findings with data from a prospectively enrolled longitudinal ICU cohort of patients with severe influenza. Serial cytokine measurements in this cohort were used to define a core pro-inflammatory serum cytokine panel. We further examined paired respiratory samples and longitudinal immune cell profiles to explore their associations with this systemic core cytokine panel. By constructing a core cytokine panel score, we assessed whether dynamic monitoring of cytokine trajectories provided greater prognostic information than single baseline assessment and clinical predictors.

## 2 Methods

### 2.1 Study design and participants

**Figure 1:**
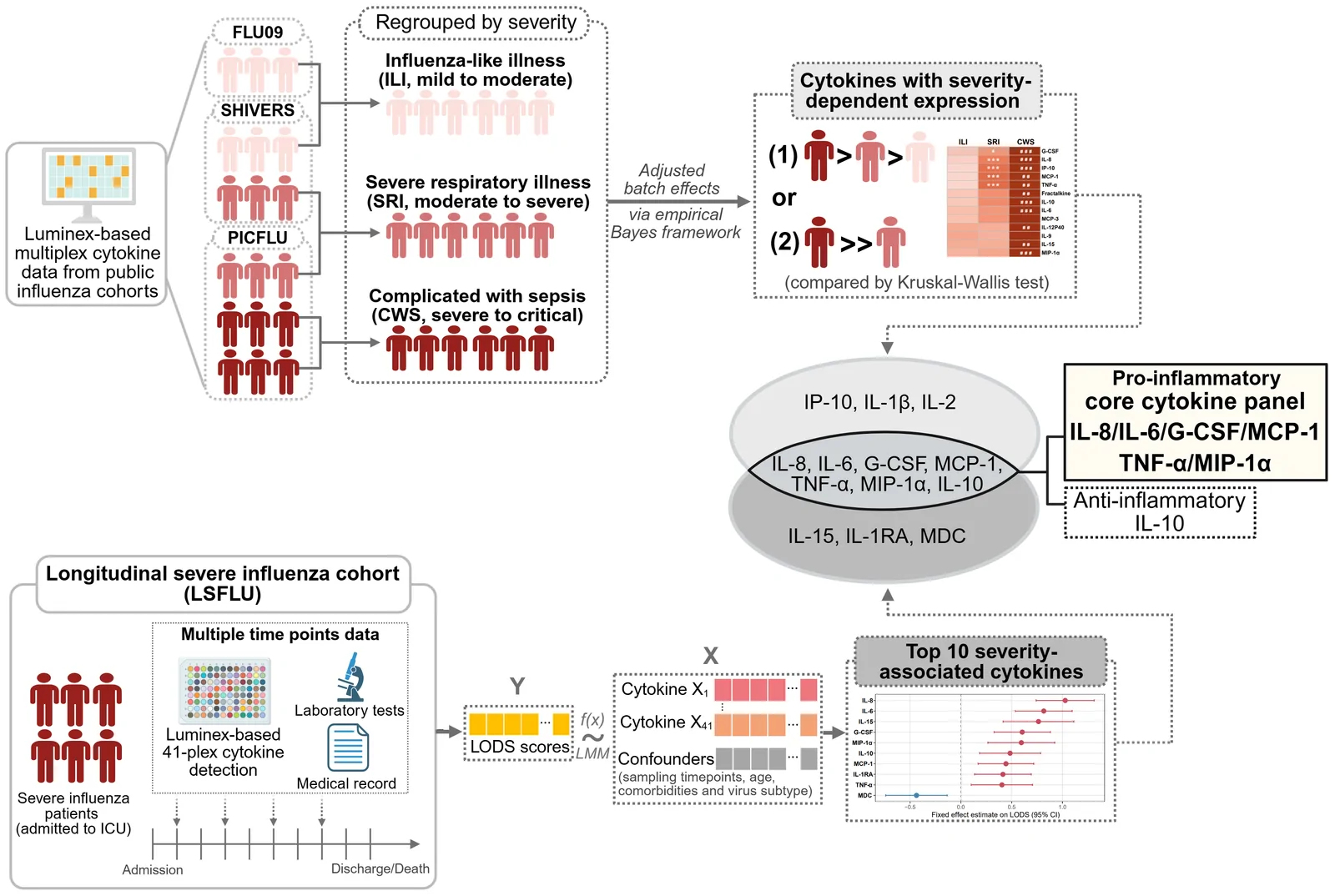
Workflow for derivation of the core pro-inflammatory cytokine panel. First, an exploratory multi-cohort analysis using publicly available data from the FLU09, SHIVERS and PICFLU studies was performed to identify cytokines showing severity-dependent expression. Second, an independent longitudinal severe influenza cohort (LSFLU) was recruited. Linear mixed models (LMM) were fitted to discover the top 10 severity-associated cytokines. Finally, two subsets of cytokines were intersected and the overlapping pro-inflammatory cytokines were defined as the core cytokine panel.

This study integrated four independent influenza cohorts to characterize cytokine signatures associated with disease severity and to evaluate their prognostic value. The overall workflow for derivation of the core pro-inflammatory cytokine panel is shown in **Figure 1**. First, we conducted an exploratory multi-cohort analysis using publicly available data from the FLU09 (9), SHIVERS (10), and PICFLU (11) studies to identify cytokines showing severity-dependent expression (12). Second, we recruited an independent longitudinal severe influenza cohort (LSFLU) to investigate the temporal dynamics of cytokines and their association with clinical outcomes, from which the top 10 severity-associated cytokines were identified. Third, the cytokines showing severity-dependent expression in the public cohorts were intersected with the top 10 severity-associated cytokines identified in the LSFLU cohort, and the overlapping pro-inflammatory cytokines were defined as the core cytokine panel. Finally, time-dependent survival analyses and landmark analyses were performed in the LSFLU cohort to assess the prognostic value of serial monitoring of this core cytokine panel.

### 2.2 Public cohorts and exploratory integrated analysis

To identify candidate cytokine signatures associated with influenza severity, we performed an exploratory integrated analysis of public influenza cohorts spanning a broad clinical severity spectrum. Public cohorts were eligible if they included serum cytokine measurements generated using a comparable multiplex assay and provided sufficient clinical metadata for harmonized severity reclassification. Based on these criteria, three public cohorts—FLU09, SHIVERS, and PICFLU—were included. Data were obtained from publicly available sources as previously described (12).

Across the three cohorts, serum samples for cytokine measurement were collected during the acute phase of illness, mostly within 10 days of symptom onset or ICU admission. Detailed information on clinical management was not uniformly available across the public datasets, and exact sampling schedules were not consistently reported. The available demographic, clinical, and enrollment-related variables are summarized in **Table 1**.

**Table 1:** Summary of available demographic and clinical characteristics across the analyzed public influenza cohorts.

Cohort (Total no. of patients)	FLU09 (N = 308)	SHIVERS (N = 87)	PICFLU (N = 221)
Analyzed no. of patients, n	27	87	72
Demographics			
Age, years [means ± SD (range)]	20.5 ± 14.9 (0-69.0)	44.4 ± 17.8 (12.0-78.0)	8.6 ± 5.2 (0-18.1)
Male Gender, n (%)	9 (33.3%)	36 (41.4%)	NA^†^
Clinical characteristics and outcome			
ICU admission, n (%)	0 (0%)	4 (4.6%)	72 (100%)
Severe respiratory illness, n (%)	0 (0%)	60 (69.0%)	72 (100%)
Sepsis, n (%)	0 (0%)	0 (0%)	57 (79.2%)
In-hospital death, n (%)	0 (0%)	0 (0%)	7 (9.7%)
DAO at study enrollment [median (IQR)]	NA^†^	4.0 (3.0-8.0)	NA^†^

^†^ NA, not applicable. Data were summarized using variables that were retrievable from the original public datasets. Not all baseline characteristics were uniformly available for three cohorts.

DAO, days after symptom onset.

FLU09 comprised subjects ranging from asymptomatic household contacts to influenza-infected patients. SHIVERS comprised two groups of influenza-infected adults, classified according to the World Health Organization case definitions (10, 13). Patients in the SHIVERS cohort were categorized as having either acute respiratory illness (ARI, outpatient) or severe acute respiratory illness (SARI, hospitalized). PICFLU enrolled children with critical influenza admitted to the ICU. Across the three cohorts, 37 cytokines were measured using the Milliplex human cytokine immunoassay (Millipore) on the Luminex platform, ensuring methodological comparability of cytokine quantification.

**Figure 2:**
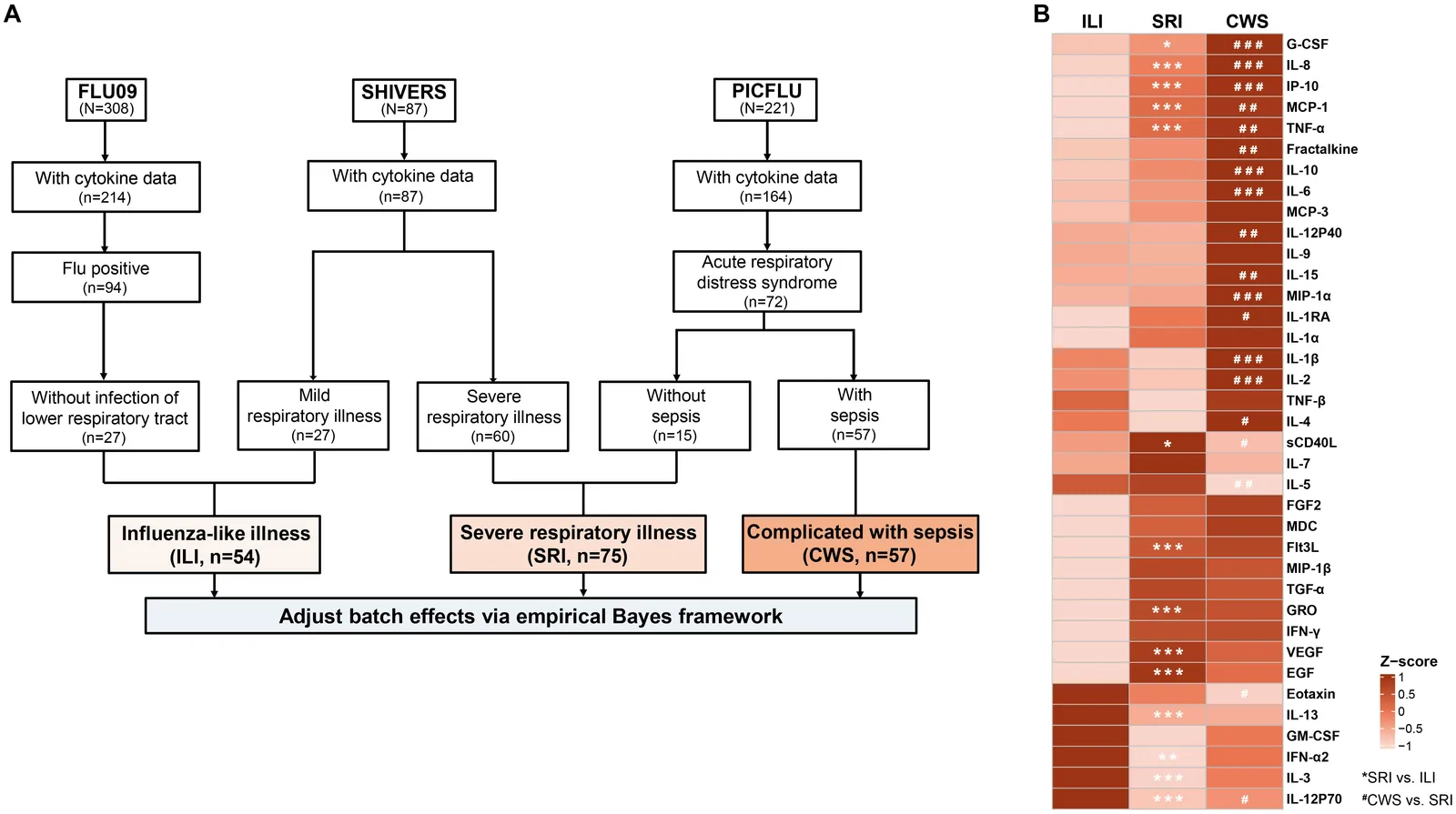
Integrated multi-cohort analysis revealed candidate cytokines with severity-dependent expression patterns. **(A)** Schematic workflow of data integration and processing. Three independent influenza cohorts (FLU09, SHIVERS, and PICFLU) were pooled. Batch effects were adjusted using an empirical Bayes framework to harmonize cytokine expression levels across datasets. Patients were categorized into three severity groups: (1) influenza-like illness (ILI, mild cases), (2) severe respiratory illness (SRI, hospitalized cases without sepsis), and (3) complicated with sepsis (CWS, critical cases). **(B)** Heatmap illustrating the relative expression levels (Z-scores) of serum cytokines across the identified severity groups. The color scale represents the normalized expression intensity (dark red: high; light red: low). Statistical significance between sequential severity stages is indicated: *denotes adjusted *P* < 0.05 for SRI vs. ILI; # denotes adjusted *P* < 0.05 for CWS vs. SRI. */#, *P* < 0.05; **/##, *P* < 0.01; ***/###, *P* < 0.001.

At the subject level, only individuals with complete serum cytokine profiles and sufficient clinical information for severity assignment were retained. Subjects were subsequently regrouped into harmonized severity categories (**Figure 2A**). In FLU09, only influenza-positive subjects without lower respiratory tract infection were included and classified as influenza-like illness (ILI). In SHIVERS, outpatient ARI cases were classified as ILI, whereas hospitalized SARI cases were classified as severe respiratory illness (SRI). In PICFLU, ICU patients without sepsis were classified as SRI, whereas those with sepsis were classified as complicated with sepsis (CWS). These categories were created as a pragmatic, harmonized severity gradient to enable exploratory comparisons across cohorts, rather than as a standardized clinical severity definition applied uniformly across all studies.

### 2.3 Longitudinal cohort

To characterize the temporal dynamics of inflammatory mediators, we prospectively recruited adult patients with laboratory-confirmed influenza, including avian influenza H7N9 and seasonal influenza, who were admitted to the ICU at The First Affiliated Hospital of Guangzhou Medical University between 2013 and 2019. Eligible patients were adults with laboratory-confirmed influenza requiring ICU admission. Patients with primary immunodeficiency or secondary immunodeficiency due to conditions such as HIV infection, active malignancy, organ transplantation, chemotherapy, or long-term immunosuppressive therapy were excluded. A total of 33 patients with severe influenza were included in this cohort, which was designated the longitudinal severe influenza cohort (LSFLU).

Clinical specimens, including serum, sputum, and peripheral blood mononuclear cells (PBMC), were collected at enrollment and serially during hospitalization. Samples were not collected at fixed prespecified intervals. However, the available longitudinal samples covered different stages of ICU stay, including an early ICU phase (within 10 days after ICU admission) and a late ICU phase (more than 20 days after ICU admission). For longitudinal analysis of linear mixed models, all available repeated serum and sputum measurements were included. In total, 176 serum samples and 179 sputum samples from 33 patients were analyzed.

The study protocol for the LSFLU cohort was approved by the Ethics Committee of The First Affiliated Hospital of Guangzhou Medical University (Ethics No. 2016-78). Written informed consent was obtained from all patients or their legal surrogates.

### 2.4 Clinical data and severity definitions

For the integrated multi-cohort analysis, disease severity was categorized into three degrees: (1) influenza-like illness (ILI), representing mild or asymptomatic cases; (2) severe respiratory illness (SRI), representing patients requiring hospitalization but not in ICU; and (3) complicated with sepsis (CWS), representing critically ill patients complicated with sepsis that require ICU admission.

For the LSFLU cohort, comprehensive clinical data were extracted from electronic medical records and reviewed by a team of experienced respiratory physicians. The disease severity was assessed using the Logistic Organ Dysfunction System (LODS) score (14, 15). The severity of lung injury were evaluated using ventilator-free days (VFD) at day 28 of hospitalization, defined as the number of days a patient survived and was free from mechanical ventilation (16).

### 2.5 Cytokine quantification

Serum and sputum samples obtained from subjects in the LSFLU cohort were stored at -80 °C and subsequently analyzed using the MILLIPLEX Human Cytokine/Chemokine Magnetic Bead Panel Premixed 41-Plex (Millipore, Cat# HCYTMAG60PMX41BK). The assay was performed on the Bio-Plex 200 Multiplex Testing System (Bio-Rad) following the manufacturer’s instructions. Cytokine concentrations were calculated from standard curves. For statistical analysis, values below the limit of detection (LOD) were imputed as LOD/2.

### 2.6 Statistical analysis

#### 2.6.1 General approach

To compare characteristics between survivors and non-survivors in the LSFLU cohort, the Student’s *t*-test was used for age. The Mann-Whitney U test was performed for non-normally distributed continuous variables, including days after ICU admission (DAA), LODS scores, ICU length of stay and VFD. Fisher’s exact test was used for categorical variables (presented as counts and percentages). All statistical analyses were performed using R software (version 4.2.2). Analyses were performed using available complete data for each model; there were no missing mortality outcomes. Clinical covariates included in the regression models were complete in the corresponding analytic datasets, and no multiple imputation was performed. A two-sided *P*-value < 0.05 was considered statistically significant.

#### 2.6.2 Integrated multi-cohort analysis

Batch effects across three cohorts (FLU09, SHIVERS, and PICFLU) were adjusted via an Empirical Bayes framework (ComBat function from the R package *sva*) (17). Comparisons of cytokine levels among three degrees of severity (ILI, SRI, and CWS) were performed using the Kruskal-Wallis test. When the global test was significant, *post-hoc* pairwise comparisons were conducted using Dunn’s test with Bonferroni correction for multiple testing. Because the aim was to derive a clinically interpretable core pro-inflammatory cytokine panel for serial monitoring, downstream candidate selection focused on cytokines showing severity-dependent upregulation, whereas cytokines with decreasing trends were described but not advanced for panel derivation (details in **Supplementary Table 1**).

#### 2.6.3 Longitudinal analysis and linear mixed models

Temporal cytokine dynamics were visualized using locally estimated scatterplot smoothing (LOESS) regression curves with 95% confidence intervals (CI). To evaluate longitudinal associations between cytokine dynamics and disease severity, linear mixed-effects models (LMM) were fitted using the *lme4* package in R. All available repeated measurements were included, and patient identity was specified as a random intercept to account for within-patient correlation due to repeated sampling. Days after ICU admission (DAA) was included as a fixed-effect covariate in all models to account for differences in sampling time.

For systemic severity analyses, LODS score was modeled as the dependent variable, and each serum cytokine was entered separately as the primary predictor after log_10_ transformation and z-score standardization, with adjustment for age, comorbidity status, DAA and virus type (H7N9 vs. seasonal influenza). Fixed-effect estimates therefore represented the change in LODS score per one-standard-deviation increase in log_10_-transformed serum cytokine level.

For lung injury severity analyses, log_10_-transformed sputum cytokine levels were modeled as the dependent variable. Patients were stratified by ventilator-free days (VFDs) into low-VFD (<7 days; more severe lung injury) and high-VFD (≥7 days; milder lung injury) groups, and each sputum cytokine was compared between groups using LMM adjusted for age, lung comorbidity status, DAA and virus type. Fixed-effect estimates for the VFD group variable were extracted to quantify adjusted between-group differences in sputum cytokine levels.

#### 2.6.4 Construction of the core cytokine panel score

As shown in **Figure 1**, the core cytokine panel was derived through a two-step strategy. This two-step design was intended to reduce the likelihood that cytokines specific to a single public cohort or demographic subgroup would determine the final panel. First, in the integrated analysis of the three public influenza cohorts, candidate cytokines were defined as upregulated cytokines showing severity-dependent expression, including either a stepwise increase across severity groups or a marked increase in the most severe group. Second, these candidate cytokines were intersected with the top 10 severity-associated cytokines identified in LSFLU cohort. The overlapping pro-inflammatory cytokines—IL-8, IL-6, G-CSF, MCP-1, TNF-α, and MIP-1α—were defined as the core cytokine panel.

Based on the six-cytokine core panel, a composite core panel score was constructed to quantify the aggregate inflammatory burden. To ensure comparability across cytokines with different physiological ranges, expression levels were standardized using Z-scores (by R package *tidyverse*). The reference mean and standard deviation (SD) for each cytokine were derived from early LSFLU samples, defined as the first sample collected within 5 days of ICU admission (**Supplementary Table 2**). For each patient at each time point, the core panel score was calculated as the arithmetic mean of the Z-scores of the six core cytokines, with higher scores indicating greater aggregate inflammatory burden.

#### 2.6.5 Time-dependent survival analysis

To evaluate the prognostic value of dynamic cytokine changes in the LSFLU cohort, survival analysis was performed using a time-varying Cox proportional hazards framework (R package *survival*). Longitudinal data were structured in a counting process format (start-stop intervals) to handle time-dependent covariates. Three distinct models were constructed to dissect the risk contribution: (1) Baseline model: incorporated only the baseline panel score at study enrollment; (2) Dynamic model: partitioned the risk into the baseline score and the delta score (defined as current score - baseline score) to independently assess the impact of initial severity versus subsequent trajectory; (3) Current model: used the time-updated panel score as a continuous time-dependent covariate. Additional time-dependent Cox models including LODS scores and C-reactive protein (CRP) and diabetes-adjusted sensitivity analyses were performed. Model performance was evaluated by comparing the Akaike Information Criterion (AIC) across models to assess relative goodness-of-fit while accounting for model complexity, and by calculating the concordance index (C-index) to quantify the discriminative ability of each model in ranking individual mortality risk over time. The proportional hazards (PH) assumption for all constructed Cox proportional hazards models was formally evaluated using the scaled Schoenfeld residuals test.

To account for repeated measures within individuals, robust variance estimation was applied using clustering by patient ID. The Simon-Makuch method was employed to visualize survival probabilities stratified by time-updated risk groups (high vs. low compared to reference).

#### 2.6.6 Landmark analysis

To assess how predictive performance evolved over the clinical course, landmark analyses were performed in the LSFLU cohort (R package *tidyverse* and *survival*). Landmark times were prespecified at days 5, 8, 11, 14, and 17 after ICU admission. Landmark time points were selected based on the distribution of sampling frequency to maximize data availability over follow-up.

At each landmark time (*t_LM_*), the analysis was restricted to patients who remained alive and under follow-up beyond that time point. For each patient, the most recent cytokine panel score available up to and including the landmark day was extracted. Two logistic regression models were then fitted to predict subsequent in-hospital mortality after the landmark day: (1) a baseline model including only the baseline panel score measured at enrollment, and (2) an updated model incorporating both the baseline score and the most recent panel score available by the landmark day (*t* ≤ *t_LM_*). Discriminative performance was assessed using the area under the receiver operating characteristic curve (AUC). Bootstrap resampling (500 iterations) was used to estimate 95% confidence intervals for the AUCs at each landmark time.

#### 2.6.7 Compartmental analysis

To investigate the compartmentalization of host response, cytokine concentrations in time-point-matched serum and sputum samples were analyzed. Paired comparisons were performed using the Wilcoxon signed-rank test. The association between systemic (serum) and local (sputum) levels was evaluated using Spearman’s rank correlation analysis.

## 3 Results

### 3.1 Integrated multi-cohort analysis identified candidate cytokines with severity-dependent expression

After harmonized severity reclassification and empirical Bayes adjustment for inter-cohort batch effects (17), the three public influenza cohorts were integrated for joint analysis. A total of 186 subjects with cytokine data and sufficient clinical information were retained and regrouped into three severity categories representing a harmonized influenza severity gradient: influenza-like illness (ILI, n=54), severe respiratory illness (SRI, n=75), and cases complicated with sepsis (CWS, n=57) (**Figure 2A**).

Comparative analysis and heatmap clustering of the serum cytokines revealed distinct expression patterns associated with increasing influenza severity (**Figure 2B**). Details of significantly changed cytokines are summarized in **Supplementary Table 1**. Some cytokines, such as sCD40L, Flt3L and VEGF, were significantly elevated in SRI compared with ILI but did not further increase in CWS, suggesting that these markers primarily reflected the transition from mild to severe respiratory illness rather than progression to critical illness. In contrast, a critical illness-associated cluster, including IL-6, IL-10 and MIP-1α, exhibited a marked surge specifically in the CWS group relative to SRI, indicating a strong association with the most severe disease state. A subset of markers, such as IL-13 and IFN-α2, displayed decreasing trends with increasing severity; however, these cytokines were not advanced for core panel derivation because the panel was specifically designed to capture pro-inflammatory escalation associated with worsening disease and to support clinically interpretable serial monitoring. Accordingly, subsequent analyses focused on upregulated cytokines.

To derive candidate cytokines from the integrated multi-cohort analysis, cytokines with severity-dependent expression were defined as those meeting either of two criteria: (1) a stepwise increase across severity groups (CWS > SRI > ILI), or (2) a marked increase in the most severe group compared with the severe respiratory illness group (CWS >> SRI, with Bonferroni-corrected *P* < 0.001). Based on these criteria, five cytokines—G-CSF, IL-8, IP-10, MCP-1, and TNF-α—showed a stepwise increase from ILI to SRI to CWS, whereas another five cytokines—IL-10, IL-6, MIP-1α, IL-1β, and IL-2—showed a marked surge in CWS relative to SRI. Together, these 10 cytokines were identified as candidate cytokines with severity-dependent expression in the integrated multi-cohort analysis (**Figure 1**). Because the integrated analysis combined adult and pediatric cohorts, these cytokines were treated as candidate markers and further filtered by intersection with the top severity-associated cytokines identified in the LSFLU cohort to derive the final core panel.

### 3.2 Clinical characteristics of the longitudinal LSFLU cohort

To capture the temporal dynamics of serum cytokines in relation to disease progression, we recruited a severe influenza cohort (LSFLU) comprising 33 patients with laboratory-confirmed influenza A virus infection requiring ICU admission. The demographics and clinical characteristics are summarized in **Table 2**. Based on the outcome, patients were categorized into survivors (n=23) and non-survivors (n=10). There were no statistically significant differences between survivors and non-survivors in age (*P* = 0.325), gender (*P* = 0.245), or the distribution of avian influenza (H7N9) versus seasonal influenza infection (*P* = 1.000), suggesting no obvious baseline imbalance in viral subtype between outcome groups. However, non-survivors exhibited a significantly higher prevalence of diabetes (40.0% vs. 4.3%, *P* = 0.021). Consistent with their fatal outcome, non-survivors presented with greater disease severity at enrollment, indicated by significantly higher LODS scores (median 4.5 vs. 3.0, *P* = 0.008) and a higher requirement for ECMO support (70.0% vs. 26.1%, *P* = 0.026). Moreover, trajectories of LODS scores suggested that, non-survivors suffered from sustained multiple organ dysfunction (**Supplementary Figure 1**). Furthermore, non-survivors had significantly lower VFD values within 28 days compared to survivors (median 0.5 vs. 7.0, *P* = 0.001), reflecting more severe and prolonged lung injury. Bacterial co-infection rates were high in both groups (>90% for respiratory tract) but showed no significant difference (*P* = 1.000), indicating that the prevalence of secondary bacterial infection per se did not distinguish non-survivors from survivors in this cohort.

**Table 2:** Demographics and clinical characteristics of the LSFLU cohort.

Variable	Survivors (n=23)	Non-survivors (n=10)	*P* value
Demographics			
Age, years [means ± SD (range)]	48.9 ± 16.0 (17-76)	54.8 ± 15.1 (31-74)	0.325
Male Gender, n (%)	12 (52.2%)	8 (80.0%)	0.245
Comorbidities			
Diabetes, n (%)	1 (4.3%)	4 (40.0%)	0.021
Hypertension, n (%)	2 (8.7%)	2 (20.0%)	0.567
Cardiovascular diseases, n (%)	2 (8.7%)	0 (0.0%)	1.000
Hepatitis, n (%)	2 (8.7%)	1 (10.0%)	1.000
Chronic lung diseases, n (%)	2 (8.7%)	1 (10.0%)	1.000
Smoking history, n (%)	4 (17.4%)	0 (0.0%)	0.289
Cancer, n (%)	2 (8.7%)	0 (0.0%)	1.000
Clinical characteristics			
DAA at study enrollment [median (IQR)]	4.0 (2.0-5.0)	6.0 (3.2-8.5)	0.098
LODS at study enrollment [median (IQR)]	3.0 (1.0-4.0)	4.5 (4.0-6.0)	0.008
Treatments and complications			
Invasive mechanical ventilation, n (%)	19 (82.6%)	10 (100.0%)	0.289
ECMO support, n (%)	6 (26.1%)	7 (70.0%)	0.026
Bacterial co-infection^†^			
Respiratory tract, n (%)	21 (91.3%)	10 (100.0%)	1.000
Bloodstream, n (%)	2 (8.7%)	3 (30.0%)	0.149
Virus type			
HPAI (H7N9) viruses, n (%)	15 (65.2%)	6 (60.0%)	1.000
Seasonal influenza viruses, n (%)	8 (34.8%)	4 (40.0%)	1.000
Outcomes			
ICU length of stay, days [median (IQR)]	24.0 (17.5-38.0)	23.5 (18.5-33.0)	0.891
Ventilator-free days^‡^ [median (IQR)]	7.0 (5.0-15.0)	0.5 (0.0-2.0)	0.001

^†^ Bacterial co-infection was confirmed by positive sputum/blood culture results.

^‡^ Ventilator-free days were defined as the number of days a patient is alive and free from mechanical ventilation within 28 days.

DAA, days after ICU admission. LODS, logistic organ dysfunction score; ECMO, extracorporeal membrane oxygenation; HPAI viruses, highly pathogenic avian influenza viruses.

### 3.3 Identification of a core pro-inflammatory cytokine panel

**Figure 3:**
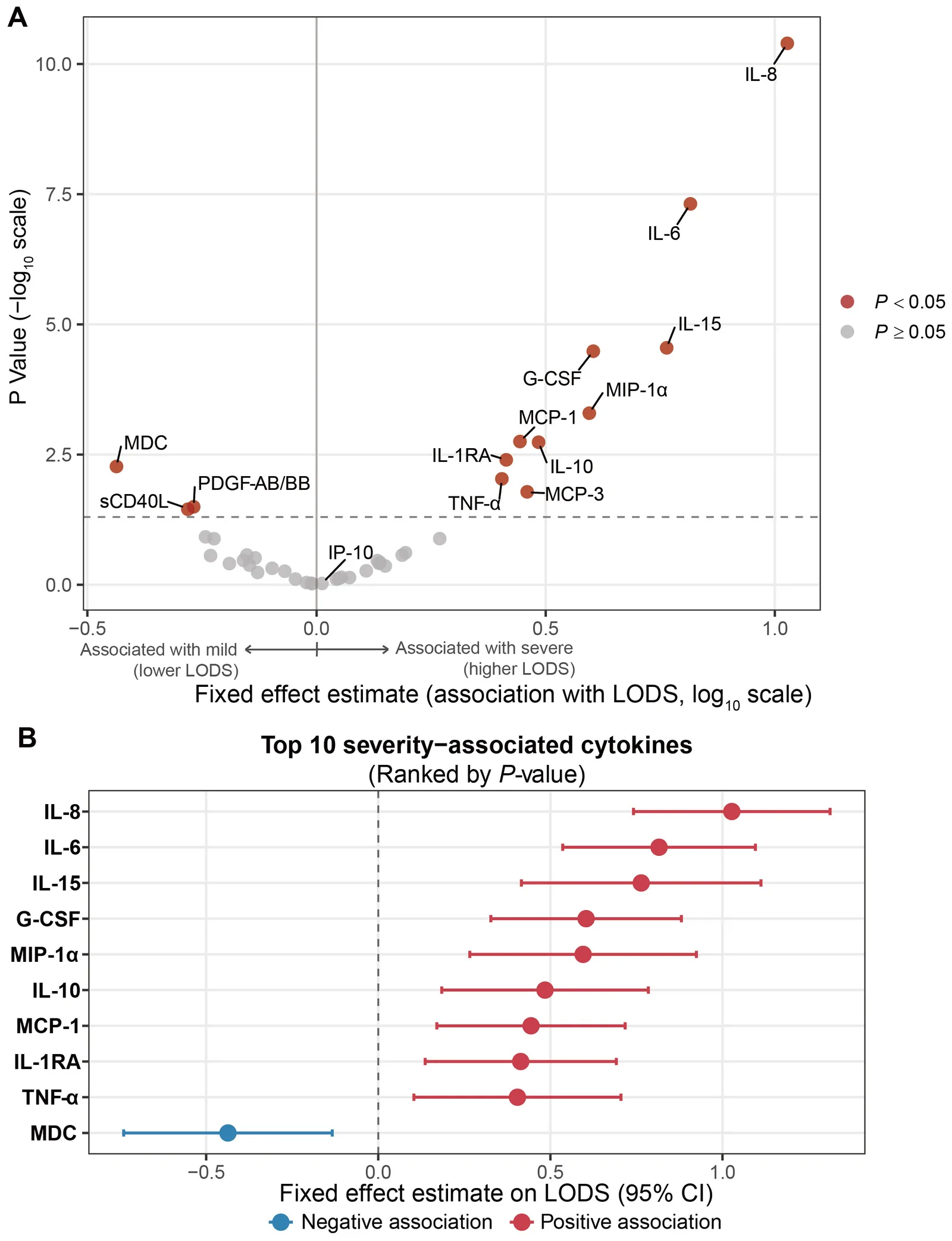
Screening and identification of cytokines associated with disease severity in the LSFLU cohort. **(A)** Volcano plot illustrating the longitudinal association between serum cytokines and disease severity (measured by LODS) in the LSFLU cohort. A linear mixed model (LMM) was employed to calculate the effect estimates, adjusting for potential confounders including sampling time points, virus type (H7N9 vs. seasonal), age, and comorbidities. The x-axis represents the fixed effect estimate (positive values indicate a risk factor for higher severity), and the y-axis represents the -log_10_(*P*-value). Red dots denote cytokines with a significant association (*P* < 0.05); grey dots denote non-significant ones. **(B)** Forest plot displaying the top 10 cytokines most significantly associated with disease severity (ranked by LMM *P*-value). The x-axis shows the fixed effect estimate with 95% CI. Red markers indicate a positive association (higher levels correlate with higher LODS), while blue markers indicate a negative association.

Next, we sought to identify cytokines associated with disease severity in the longitudinal LSFLU cohort. Linear mixed-effects models (LMM) analysis revealed a broad spectrum of cytokines significantly associated with severity (**Figure 3A**). The top 10 severity-associated cytokines ranked by statistical significance were IL-8, IL-6, IL-15, G-CSF, MIP-1α, IL-10, MCP-1, IL-1RA, TNF-α, and MDC (**Figure 3B**). A detailed functional characterization of these severity-associated cytokines is provided in **Supplementary Table 1**.

To derive a clinically interpretable core cytokine panel, we then integrated evidence from the integrated multi-cohort analysis and the LSFLU cohort. As described above, the integrated multi-cohort analysis identified 10 candidate cytokines with severity-dependent upregulation, whereas the LMM analysis in LSFLU identified the top 10 severity-associated cytokines. Intersecting these two sets yielded seven overlapping cytokines, comprising six pro-inflammatory cytokines (IL-8, IL-6, G-CSF, MCP-1, TNF-α, and MIP-1α) and one anti-inflammatory cytokine (IL-10). Because the panel was specifically intended to capture the escalating inflammatory burden associated with worsening disease, the six pro-inflammatory cytokines were defined as the core cytokine panel (**Figure 1**).

### 3.4 Longitudinal serum trajectories and pulmonary expression patterns of the core cytokine panel

**Figure 4:**
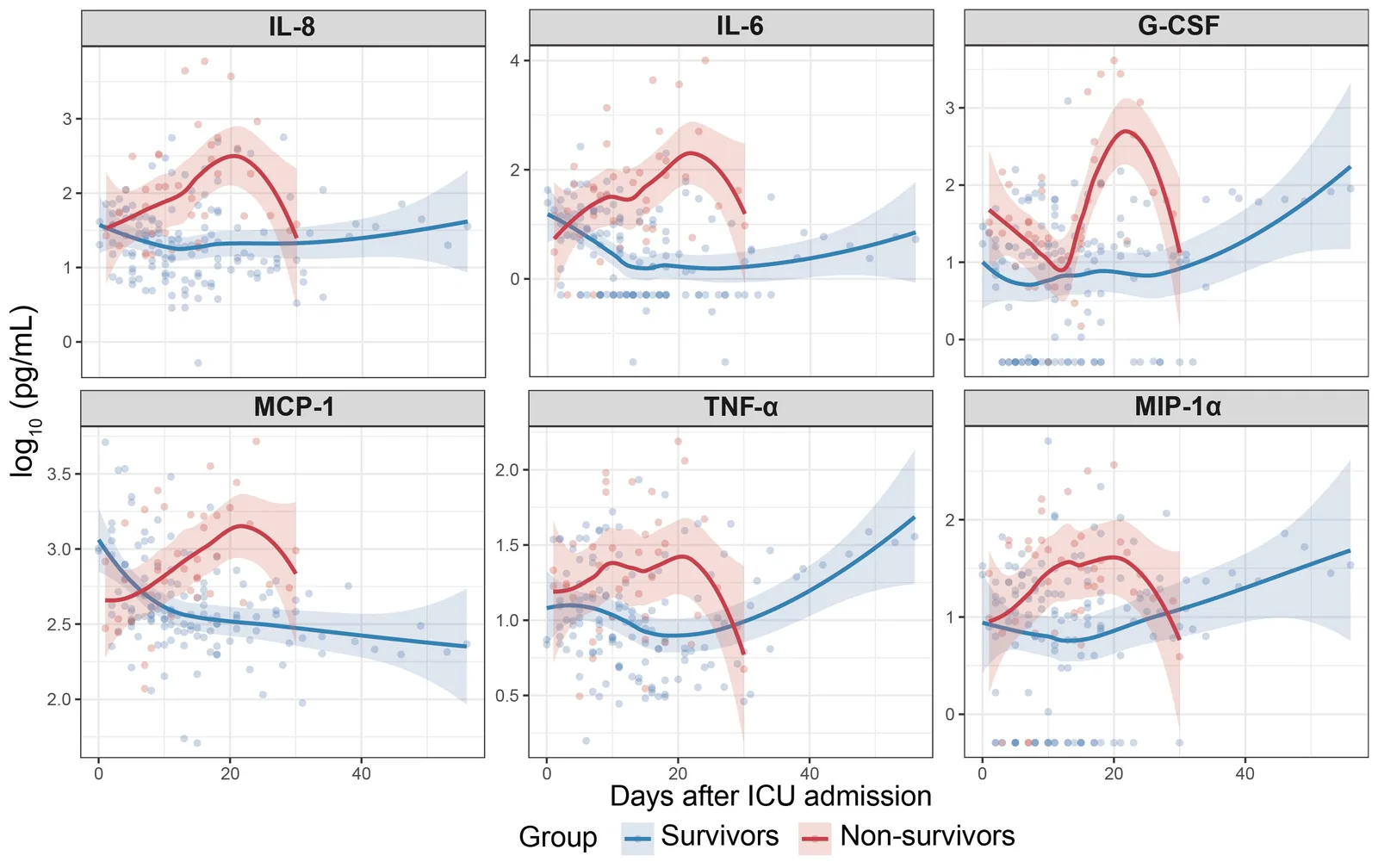
Divergent longitudinal trajectories of the core cytokine panel distinguish survivors from non-survivors. Longitudinal kinetics of the six core cytokines (IL-8, IL-6, G-CSF, MCP-1, TNF-α, and MIP-1α) stratified by clinical outcome (survivors vs. non-survivors) in the LSFLU cohort. Data points represent log_10_-transformed cytokine levels collected at multiple time points from ICU admission. Solid lines indicate the locally estimated scatterplot smoothing (LOESS) regression curves, and shaded areas represent the 95% CI. Red indicates the non-survivors group (n=10); blue indicates the survivors group (n=23).

To further elucidate the kinetics of this core panel throughout disease progression, we stratified the LSFLU cohort by clinical outcome and mapped the temporal trajectories (**Figure 4**). The analysis revealed divergent dynamic patterns between the two groups. Survivors typically exhibited a pattern of resolution, characterized by a gradual decline or maintenance of low cytokine levels during the recovery phase. In contrast, non-survivors showed a distinct biphasic trajectory: an initial phase of sustained elevation or progressive surge peaking around 2–3 weeks after ICU admission, often followed by a decline in the terminal phase. Notably, this persistent elevation (particularly evident in IL-8, IL-6, G-CSF, and MCP-1) clearly distinguished non-survivors from survivors. Such distinct divergence was less pronounced or absent in the longitudinal profiles of other measured cytokines (**Supplementary Figure 2**). Furthermore, these persistent inflammatory trajectories aligned with the sustained organ failure observed in fatal cases (**Supplementary Figure 1**), suggesting that the failure to timely resolve the pro-inflammatory response is a hallmark of poor prognosis.

**Figure 5:**
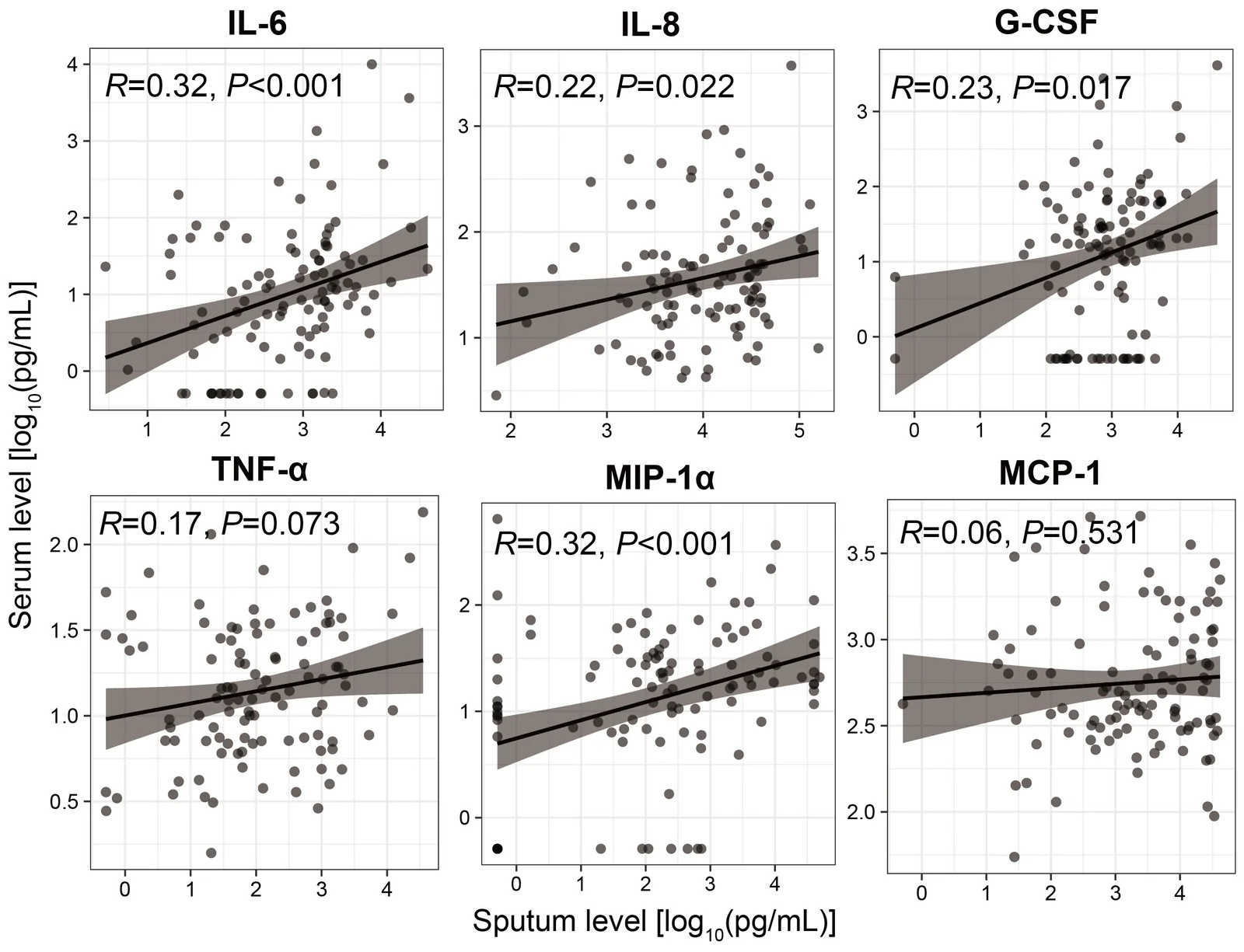
Serum core cytokine panel partially reflected pulmonary inflammation. Correlation analysis of core cytokine levels between serum and sputum collected at matched time points (28 patients, n=107 pairs). Scatter plots illustrating the relationship between serum and sputum concentrations for each cytokine. Spearman’s rank correlation coefficients (R) and *P*-values are displayed.

To determine whether these systemic signatures reflected local pulmonary inflammation, we further analyzed paired sputum samples from the same cohort. Longitudinal analysis of sputum samples (**Supplementary Figure 3**) showed that most cytokines of the identified core panel were elevated in patients with more severe lung injury, as indicated by fewer ventilator-free days. Spearman correlation analysis demonstrated modest but statistically significant associations between serum and sputum levels for several core cytokines, including IL-6, IL-8, G-CSF, and MIP-1α, whereas correlations for TNF-α and MCP-1 did not reach statistical significance (**Figure 5**). Notably, concentrations of all six core cytokines were substantially higher in sputum than in matched serum samples (**Supplementary Figure 4**). Together, these findings support a pulmonary contribution to the observed systemic cytokine signatures, with circulating levels partially reflecting the intensity of local lung inflammation. Given the practical advantages and broader clinical feasibility of blood sampling, subsequent analyses focused on evaluating the prognostic utility of the serum core cytokine panel.

### 3.5 Composite scoring of the core cytokine panel and its association with immune cell subsets

**Figure 6:**
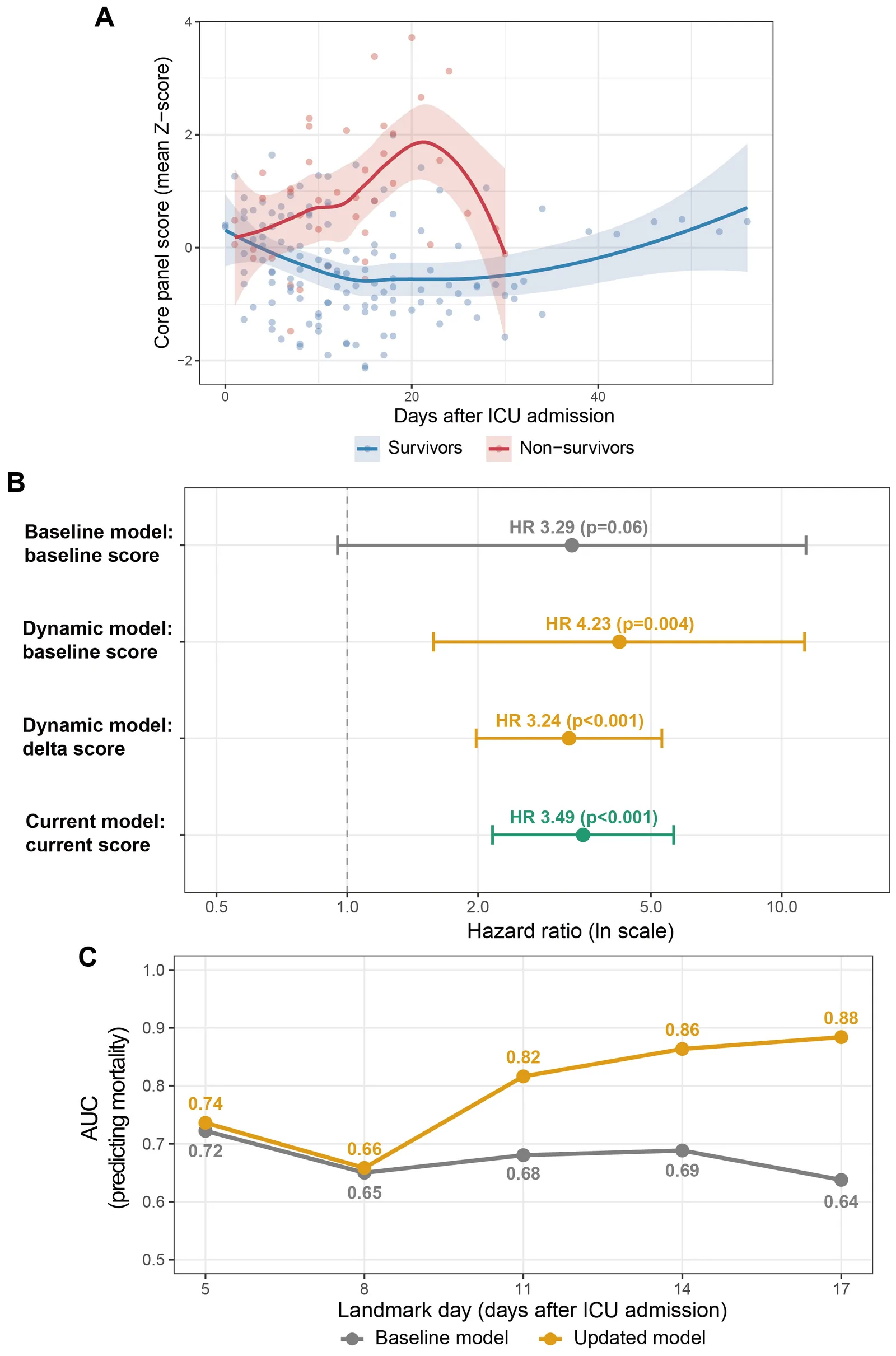
Dynamic assessment of the core cytokine panel improved mortality prediction in the LSFLU cohort. **(A)** Longitudinal trajectory of the core panel score. Dots represent individual core panel scores after ICU admission. Core panel scores were calculated as the mean Z-score of the six core cytokines relative to the reference listed in **Supplementary Table 2**. Solid lines show the LOESS regression curves, illustrating temporal trends in non-survivors (red) and survivors (blue). Shaded areas indicate 95% confidence intervals. **(B)** Hazard ratios from baseline and time-varying Cox regression models. The forest plot presents the hazard ratios (HR) and 95% CI for in-hospital mortality derived from three distinct modeling approaches. The x-axis represents HRs on a logarithmic scale. The baseline model incorporated only baseline core panel scores. The dynamic model decomposed the risk into the baseline score (fixed effect) and the delta score (time-dependent change from baseline). The current model used the time-updated current panel score as the sole predictor. **(C)** Evolution of discriminatory performance assessed by landmark analysis. The plot shows the area under the receiver operating characteristic curve (AUC) at landmark time points of day 5, 8, 11, 14, and 17 after ICU admission. The gray line represents the baseline model using only baseline cytokine data, whereas the yellow line represents the updated model incorporating the most recent cytokine data available up to each landmark day. Numbers at risk and subsequent events at each landmark time point are shown in **Table 5**.

To quantify the inflammatory burden represented by the six core cytokines, we constructed a composite core panel score by calculating the arithmetic mean of their Z-scores, using the reference mean and SD for each cytokine shown in **Supplementary Table 2**. As shown in **Figure 6A**, longitudinal trajectories of the core panel score differed markedly between survivors and non-survivors. Non-survivors exhibited progressively increasing scores over time, whereas survivors maintained consistently lower levels throughout follow-up. This divergence suggested that longitudinal monitoring of the core panel score may capture clinically relevant changes over the disease course.

We next examined whether the core panel score was associated with changes in circulating immune cell composition. In paired samples from 11 LSFLU patients, myeloid dendritic cells, plasmacytoid dendritic cells, intermediate monocytes, and non-classical monocytes increased significantly from the early to the late ICU phase (**Supplementary Figure 5A**). Overall, correlation analysis indicated that the core cytokine panel was most closely related to myeloid-lineage cells, with divergent patterns across different subsets (**Supplementary Figure 5B**). Myeloid dendritic cells and plasmacytoid dendritic cells were negatively correlated with IL-6, MCP-1, and TNF-α, whereas non-classical and intermediate monocytes showed a trend toward positive correlations with the core panel score. Together, these results provided cellular-level support that the core cytokine panel was associated with immune dysregulation rather than isolated soluble mediator changes, emphasizing the biological basis for its prognostic value.

### 3.6 Dynamic monitoring of the core cytokine panel provided additional prognostic information beyond baseline assessment

We next compared baseline and time-dependent Cox regression models using the core panel score to assess whether dynamic monitoring provided additional prognostic information beyond baseline assessment alone (**Table 3**, **Figure 6B**). Proportional hazards diagnostics did not indicate violation of the PH assumption for these models (*P*>0.05, **Supplementary Table 3**).

**Table 3:** Comparison of primary baseline and time-dependent Cox regression models for the core cytokine panel.

Model	Variable	HR (95% CI)	*P* value	C-index	AIC
Baseline model	Baseline panel score	3.29 (0.95-11.38)	0.059	0.67	34.85
Dynamic model	Baseline panel score	4.23 (1.58-11.29)	0.004	0.89	29.34
	Delta panel score	3.24 (1.98-5.30)	2.99×10^-6^		
Current model	Current panel score	3.49 (2.16-5.64)	3.37×10^-7^	0.90	27.73

HR, hazard ratio. CI, confidence interval. AIC, Akaike Information Criterion. C-index, concordance index.

In the baseline model, the baseline panel score showed only a borderline association with mortality risk (HR 3.29, 95% CI 0.95-11.38, *P* = 0.059). Incorporating longitudinal information was associated with better model fit, with the AIC decreasing from 34.85 in the baseline model to 29.34 in the dynamic model. In the dynamic model, both the baseline score (HR 4.23, 95% CI 1.58-11.29, *P* = 0.004) and the subsequent change in score (delta score: HR 3.24, 95% CI 1.98-5.30, *P* = 2.99×10^-6^) were independently associated with mortality. Among the three Cox regression models, the current model, which used the time-updated panel score (i.e., current panel score) as the sole time-dependent covariate, showed a strong association with mortality (HR 3.49, 95% CI 2.16-5.64, *P* = 3.37×10^-7^) and yielded the highest apparent C-index (0.90). These findings suggested that dynamic monitoring of the core cytokine panel may provide additional prognostic information beyond baseline assessment alone.

We examined whether influenza subtype (seasonal influenza vs. H7N9) modified the association between the panel score and mortality via interaction analyses (**Supplementary Table 4**). No significant effect modification by virus subtype was observed for either the baseline panel score (*P* = 0.222) or the current panel score (*P* = 0.366), although the power to detect interactions was limited by the modest sample size.

To visualize the influence of panel score trajectories on long-term survival, we generated time-dependent survival curves using the Simon-Makuch method (**Supplementary Figure 6**). These curves showed that patients in the low-risk group, defined by time-updated panel scores, appeared to have higher survival probabilities than those in the high-risk group.

### 3.7 Comparison with clinical predictors and diabetes-adjusted sensitivity analyses

Next, we compared the prognostic performance of the core cytokine panel with that of conventional clinical predictors, including LODS scores and CRP (**Table 4**). Current LODS was significantly associated with mortality (HR 1.44, 95% CI 1.12-1.83, *P* = 0.004), whereas baseline LODS was not (HR 1.16, 95% CI 0.88-1.53, *P* = 0.290). For CRP, the comparison was based on the baseline value available in the LSFLU cohort. Baseline CRP was not significantly associated with mortality in the Cox model (HR 1.01, 95% CI 1.00-1.02, *P* = 0.138). Compared with current LODS, the current panel score was associated with a higher apparent C-index and lower AIC.

**Table 4:** Comparison of the cytokine panel with conventional clinical predictors and diabetes-adjusted sensitivity analyses.

Model	Variable	HR (95% CI)	*P* value	C-index	AIC
Section A. Conventional predictor comparison					
Baseline panel score	Baseline panel score	3.29 (0.95-11.38)	0.059	0.67	34.85
Current panel score	Current panel score	3.49 (2.16-5.64)	3.37×10^-7^	0.90	27.73
Baseline LODS	Baseline LODS	1.16 (0.88-1.53)	0.290	0.69	38.59
Current LODS	Current LODS	1.44 (1.12-1.83)	0.004	0.88	31.52
Baseline CRP	Baseline CRP	1.01 (1.00-1.02)	0.138	0.77	29.27
Current LODS +Current panel score	Current LODS	1.18 (0.87-1.60)	0.296	0.89	28.91
	Current panel score	2.63 (1.43-4.82)	0.002		
Section B. Diabetes-adjusted sensitivity analyses					
Baseline panel score +Diabetes	Baseline panel score	2.65 (0.59-11.97)	0.205	0.68	35.25
	Diabetes	3.05 (0.81-11.44)	0.098		
Current panel score +Diabetes	Current panel score	4.24 (2.02-8.89)	1.29×10^-4^	0.92	26.24
	Diabetes	5.71 (1.21-26.97)	0.028		
Current LODS +Diabetes	Current LODS	1.41 (1.06-1.87)	0.018	0.86	31.68
	Diabetes	3.20 (0.82-12.40)	0.093		
Current LODS +Current panel score +Diabetes	Current LODS	1.14 (0.81-1.60)	0.457	0.89	27.79
	Current panel score	3.39 (1.56-7.37)	0.002		
	Diabetes	5.25 (1.19-23.14)	0.028		

HR, hazard ratio. CI, confidence interval. AIC, Akaike Information Criterion. C-index, concordance index.

We further assessed whether the core cytokine panel provided prognostic information beyond LODS. In the combined model including current LODS and the current panel score, the current panel score remained independently associated with mortality (HR 2.63, 95% CI 1.43-4.82, *P* = 0.002), whereas current LODS was no longer significant (HR 1.18, 95% CI 0.87-1.60, *P* = 0.296). These results suggested that time-updated cytokine panel scores retained prognostic association after accounting for LODS scores.

Because diabetes was more common among non-survivors than among survivors in the LSFLU cohort, we then performed diabetes-adjusted sensitivity analyses to examine whether pre-existing diabetes confounded the association between the panel score and mortality (**Table 4**, **Supplementary Figure 7**). We first compared cytokine panel scores among non-survivors stratified by diabetes status. Baseline panel scores did not differ significantly between non-survivors with and without diabetes (Mann-Whitney U test, *P* = 0.114, **Supplementary Figure 7A**). Longitudinal trajectories of the panel score were also broadly similar between the two groups, without clear separation over time (**Supplementary Figure 7B**). Moreover, in the Cox regression model adjusted for diabetes, the current panel score remained significantly associated with mortality (HR 4.24, 95% CI 2.02-8.89, *P* = 1.29×10^-4^), although these diabetes-adjusted sensitivity analyses should be interpreted cautiously given the limited sample size and evidence of non-proportionality in some models (**Supplementary Table 3**). In the model including current LODS, current panel score, and diabetes, the current panel score also remained independently associated with mortality (HR 3.39, 95% CI 1.56-7.37, *P* = 0.002), whereas current LODS was not significant (HR 1.14, 95% CI 0.81-1.60, *P* = 0.457). Although diabetes itself remained associated with mortality in panel-based adjusted models, adjustment for diabetes did not materially attenuate the association between the current panel score and mortality.

### 3.8 Updated core panel scores suggested increasing prognostic discrimination over the course of severe influenza

To further explore the potential added value of dynamic core panel evaluation, we performed landmark analyses at days 5, 8, 11, 14, and 17 after ICU admission (**Figure 6C**, **Supplementary Figure 8**, **Table 5**). At each landmark time, the baseline model used only the baseline core panel score, whereas the updated model incorporated both the baseline score and the most recent core panel score available up to that landmark day. Receiver operating characteristic curves and corresponding classification metrics at each landmark time were shown in **Supplementary Figure 8**. During the early phase after ICU admission, the two models showed similar discriminative performance, with only minimal differences in AUC at day 5 (0.72 vs. 0.74) and day 8 (0.65 vs. 0.66). At later landmark times, the AUC of the baseline model tended to decline, from 0.72 at day 5 to 0.64 at day 17. In contrast, the updated model showed a slight decrease at day 8 but subsequently showed higher AUC estimates, reaching 0.88 at day 17. Accordingly, the difference in AUC (delta AUC) between the updated and baseline models increased over time, from near zero at early landmark days to 0.24 at day 17. Together, these findings suggested that time-updated core panel scores may provide additional prognostic information over baseline assessment as severe influenza progressed, although these landmark analyses should be considered exploratory given the limited number of events at later time points.

**Table 5:** Results of the landmark analysis in the LSFLU cohort.

Landmark day^†^	No. at risk^††^	No. of events^††^	AUC^†††^ of baseline model (95% CI)	AUC^†††^ of updated model (95% CI)	Delta AUC
5	22	4	0.72 (0.49-0.94)	0.74 (0.44-1.00)	0.02
8	26	6	0.65 (0.35-0.92)	0.66 (0.35-0.94)	0.01
11	28	7	0.68 (0.40-0.93)	0.82 (0.61-0.96)	0.14
14	29	7	0.69 (0.44-0.92)	0.86 (0.67-0.97)	0.17
17	29	6	0.64 (0.36-0.91)	0.88 (0.72-0.99)	0.24

^†^ At each landmark day, analyses were restricted to patients who remained under follow-up beyond that time point.

^††^ No. at risk, referred to the number of patients eligible for prediction at the landmark time; No. of events, referred to the number of subsequent in-hospital deaths among those patients.

^†††^ AUC confidence intervals were estimated using bootstrap resampling (500 iterations).

## 4 Discussion

In this multi-cohort study, we integrated public influenza datasets with an independent longitudinal severe influenza cohort to derive a core panel of six pro-inflammatory cytokines (IL-8, IL-6, G-CSF, MCP-1, TNF-α, and MIP-1α) associated with disease severity and mortality. In the LSFLU cohort, survivors and non-survivors showed progressively greater separation in core panel scores over time, with consistently higher scores in non-survivors. Paired sputum analyses suggested that elevated serum levels of these cytokines were associated with pulmonary inflammatory activity, indicating that serum measurements may partially reflect lung inflammation. The panel was also associated with dysregulated immune cell subsets. Moreover, dynamic assessment of core panel scores remained associated with mortality after adjustment for LODS and diabetes, although some diabetes-adjusted sensitivity models should be interpreted cautiously because of evidence of non-proportionality. Compared with baseline assessment alone, time-updated panel scores were associated with higher AUC estimates at later landmark time points; however, these findings should be considered exploratory given the limited number of events.

These results underscore the pivotal role of cytokines in the diagnosis and stratification of severe influenza. The complexity of the host response extends beyond these pro-inflammatory drivers. As detailed in **Supplementary Table 1**, the cytokine profile of severe influenza involves distinct functional clusters: (1) myeloid hyperactivation, marked by the surge of neutrophil and monocyte mobilizers (e.g., IL-8, G-CSF) (18); (2) dysregulated antiviral response, marked by elevated IL-15 (a T/NK cell growth factor) alongside suppressed Th1 responses (19); (3) ineffective anti-inflammatory feedback, marked by high levels of IL-10 and IL-1RA that fail to resolve inflammation (20); and (4) impaired repair, marked by the suppression of platelet and tissue-repair factors like sCD40L and PDGF (21). This combination points to a state of systemic immune dissonance in the critical state of influenza. Notably, bacterial co-infections are frequent in severe influenza and are recognized contributors to disease progression (22). Accordingly, the observed cytokine dysregulation should not be interpreted as being exclusively attributable to influenza virus infection, because inflammatory trajectories may also be shaped by bacterial co-infection.

Crucially, our findings highlight the evolving paradigm of cytokine analysis: shifting from static biomarkers to dynamic diagnostic tools. However, current analytical approaches—relying largely on static thresholds or simple linear trends—may underserve the complexity of the host response. Beyond conventional static analyses, longitudinal cytokine measurements offer opportunities for more sophisticated modeling of host immune dynamics. Future studies could apply advanced techniques, such as signal-processing (23) or functional data analysis (24), to better distinguish persistent pathological signals from transient fluctuations in inflammatory trajectories. The superior prognostic performance of serial monitoring observed in this study supports further exploration of these approaches.

Mechanistically, the pronounced gradient observed between sputum and serum cytokine levels was consistent with the lung serving as the primary site of inflammatory amplification in severe influenza. This compartmentalized pattern aligns with current models of influenza pathogenesis, in which viral replication in alveolar epithelial cells induces highly inflammatory forms of cell death (25). These processes release damage-associated molecular patterns and pro-inflammatory mediators, generating a localized pulmonary hyperinflammatory milieu that may subsequently extend into the systemic circulation. In principle, the initial cytokine release recruits immune cells to the site of infection and promotes clearance of infected cells. During the early phase of influenza infection, local and circulating myeloid cells provide a rapid, nonspecific first-line response. However, in the setting of high viral load or pre-existing conditions that impair immune homeostasis, insufficient virus-specific responses and excessive myeloid activation can amplify tissue injury through uncontrolled pro-inflammatory cytokine release (26). The vicious cycle of cytokine release generates the cytokine storm in severe influenza patients (27). In this context, the circulating core cytokine panel may reflect the magnitude of virus-induced pulmonary injury and its systemic inflammatory consequences.

Notably, longitudinal trajectories among non-survivors demonstrated a biphasic pattern, characterized by an early marked inflammatory elevation followed by a terminal decline. This temporal evolution is compatible with the concept that critical influenza may involve distinct immunological phases, including an initial hyperinflammatory endotype that may transition toward immune dysfunction or immunoparalysis in advanced disease (28). Such dynamics are consistent with the recently proposed “tipping point” framework (29), which posits that early inflammatory surges can drive irreversible structural damage once a critical threshold is exceeded. The emerging success of immune endotype-guided strategies in sepsis (30) further underscores the potential relevance of immunological heterogeneity in severe influenza. Failure to account for dynamic immune states may partly explain the inconsistent results of prior immunomodulatory trials (7), as interventions beneficial during early hyperinflammation could become ineffective or harmful once immune responses shift toward suppression.

Collectively, these findings support a shift toward more individualized approaches to immunomodulation in severe influenza, rather than uniform treatment strategies. Serial immune monitoring may provide a means to capture evolving immune states and facilitate patient stratification based on dominant inflammatory endotypes. Current clinical guidelines remain cautious regarding corticosteroid use in influenza (31), although recent trials have demonstrated mortality benefits of low-dose corticosteroids in severe community-acquired pneumonia (32). Given the lack of definitive evidence for novel anti-inflammatory agents (6, 33) in large-scale influenza trials, these observations underscore the potential importance of treatment timing. Accordingly, the identified core cytokine panel may serve as a prognostic and hypothesis-generating tool to inform future studies exploring the alignment of immunomodulatory interventions with dynamic inflammatory trajectories.

The inclusion of adult and pediatric cohorts, as well as seasonal and avian influenza cases in the LSFLU cohort, should be viewed in light of the study objective—to identify a cytokine panel applicable across heterogeneous severe influenza settings rather than one specific to a single age group or viral subtype. Recent integrative analyses showed that although cytokine profiles varied by age, several inflammatory mediators, including IL-6, IL-8, G-CSF, and TNF-α, were consistently associated with severe influenza across different populations (34). Similarly, previous studies reported overlapping elevations of IL-6, IL-8, and MCP-1 in severe cases of both seasonal and avian influenza (35). In this context, our panel may represent a shared inflammatory signature across diverse influenza presentations, although age-specific and subtype-specific immune responses may still exist and warrant further investigation.

Our study had several limitations. First, the longitudinal cohort was relatively small and included a limited number of deaths, which restricted the events-per-variable ratio for multivariable survival analyses. As a result, some hazard ratio estimates were imprecise, and the risk of overfitting cannot be excluded, particularly in sensitivity analysis. Second, the observational design and retrospective analyses may have introduced selection bias. Third, the analysis was restricted to a predefined panel of cytokine measurement. Although bacterial co-infection rates were similarly high across outcome groups, detailed data on the timing, severity, and treatment response of co-infections were unavailable; therefore, residual confounding by bacterial co-infection and other unmeasured factors cannot be excluded. Fourth, while most Cox models satisfied the proportional hazards assumption, some diabetes-adjusted sensitivity models showed evidence of non-proportionality, suggesting that the effect of diabetes may vary over time. Fifth, event numbers at individual landmark time points were limited. Our predictive analyses should therefore be interpreted as exploratory and hypothesis-generating, intended to identify potentially informative cytokine signals rather than to define a finalized prognostic model. Formal calibration assessment and internal validation were not performed because the limited sample size and small number of outcome events would likely have produced unstable estimates. Robust external validation was not feasible because comparable influenza cohorts with serial cytokine measurements remained scarce. Its generalizability across centers, populations and clinical settings therefore remained unclear. Prospective studies with larger cohorts, standardized cytokine assays, and broader immune profiling are needed to validate and refine these findings.

In summary, this multi-cohort study identified a six-cytokine serum panel associated with influenza severity and mortality. Longitudinal analyses revealed divergent inflammatory trajectories between survivors and non-survivors, dynamic monitoring suggesting incremental prognostic value over baseline assessment. These findings highlight the potential value of longitudinal inflammatory profiling and warrant prospective validation in larger cohorts to define its role in the precision management of severe influenza.

## Data Availability

The datasets presented in this article are not readily available because the LSFLU datasets are not publicly available due to ethical and privacy considerations, but are available from the corresponding author upon reasonable request. Data from the FLU09, SHIVERS, and PICFLU cohorts are openly accessible from their original repositories. Requests to access the datasets should be directed to ZY (email: jeffyah@163.com).
