# Supplementary material for: Dynamic monitoring of a core pro-inflammatory cytokine panel improves mortality prediction in severe influenza
Source: Front Immunol. 2026 Aug 19;17:1805650. doi: 10.3389/fimmu.2026.1805650 (PMC13533885; doi:10.3389/fimmu.2026.1805650)
Supplement: Supplementary file 1 [file DataSheet1.pdf]

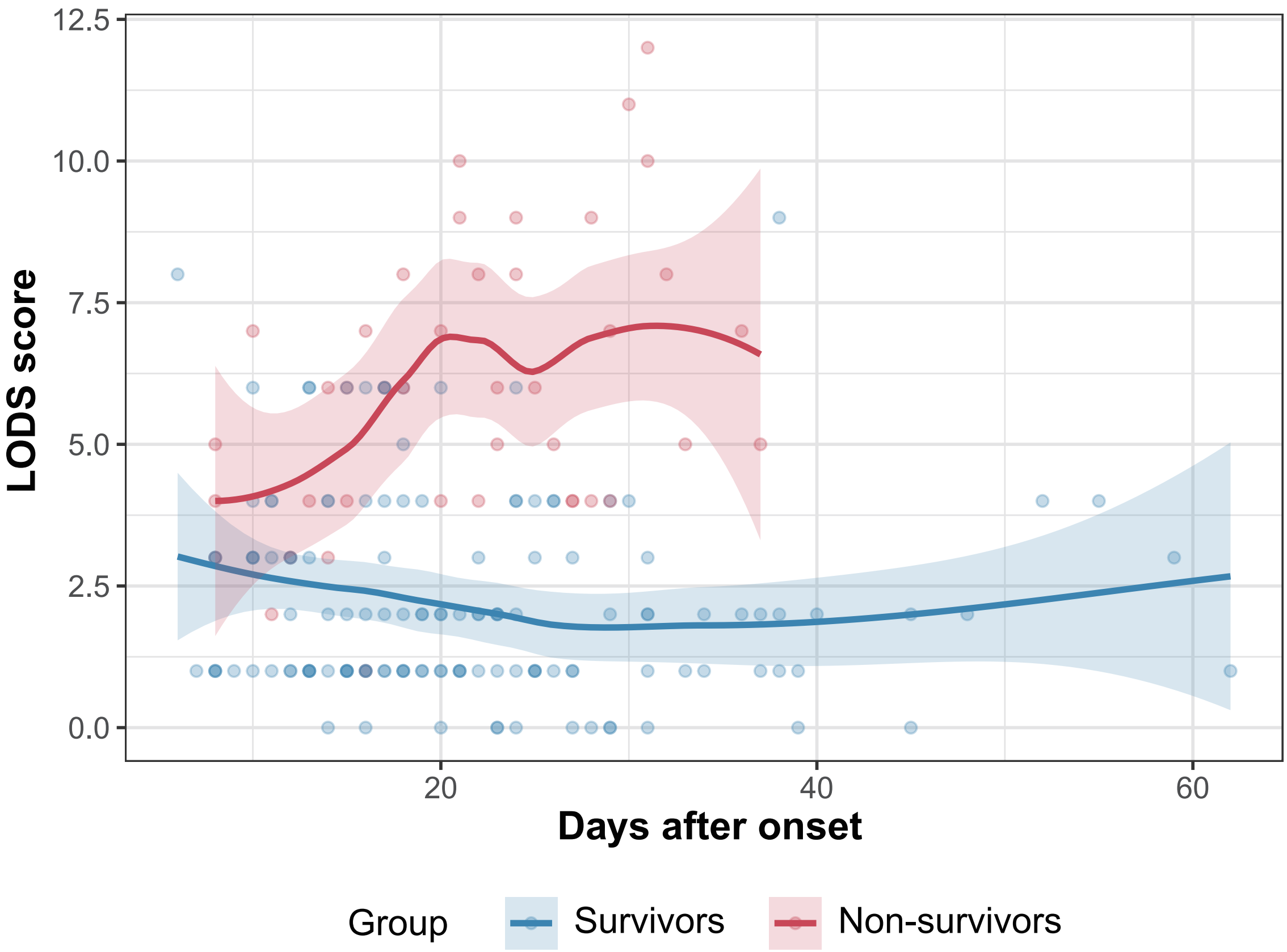

Figure S1. Longitudinal trajectories of LODS scores in LSFLU cohort.

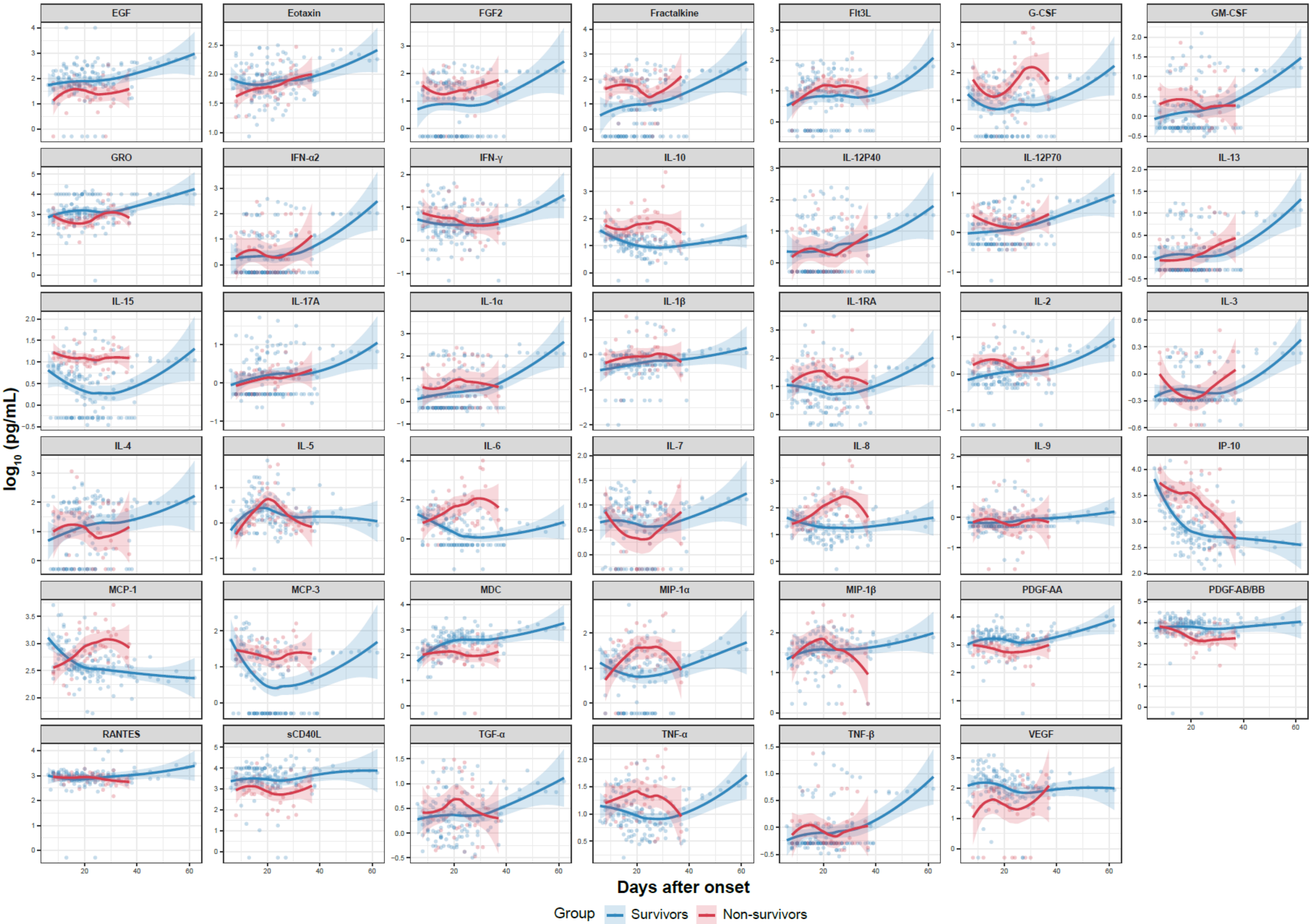

Figure S2. Longitudinal trajectories of serum cytokines in the LSFLU cohort.

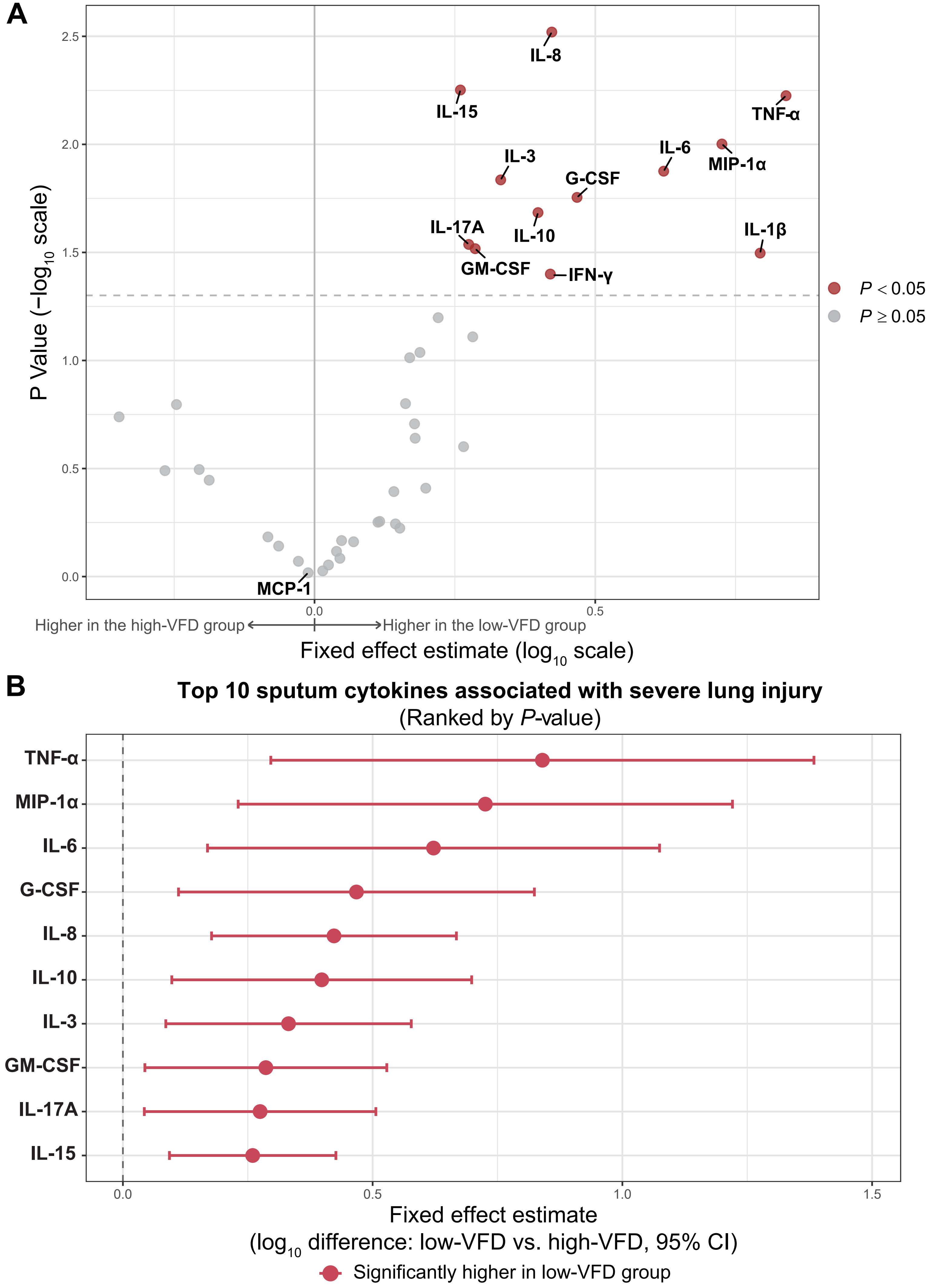

Figure S3. Sputum cytokines longitudinally associated with lung injury severity.

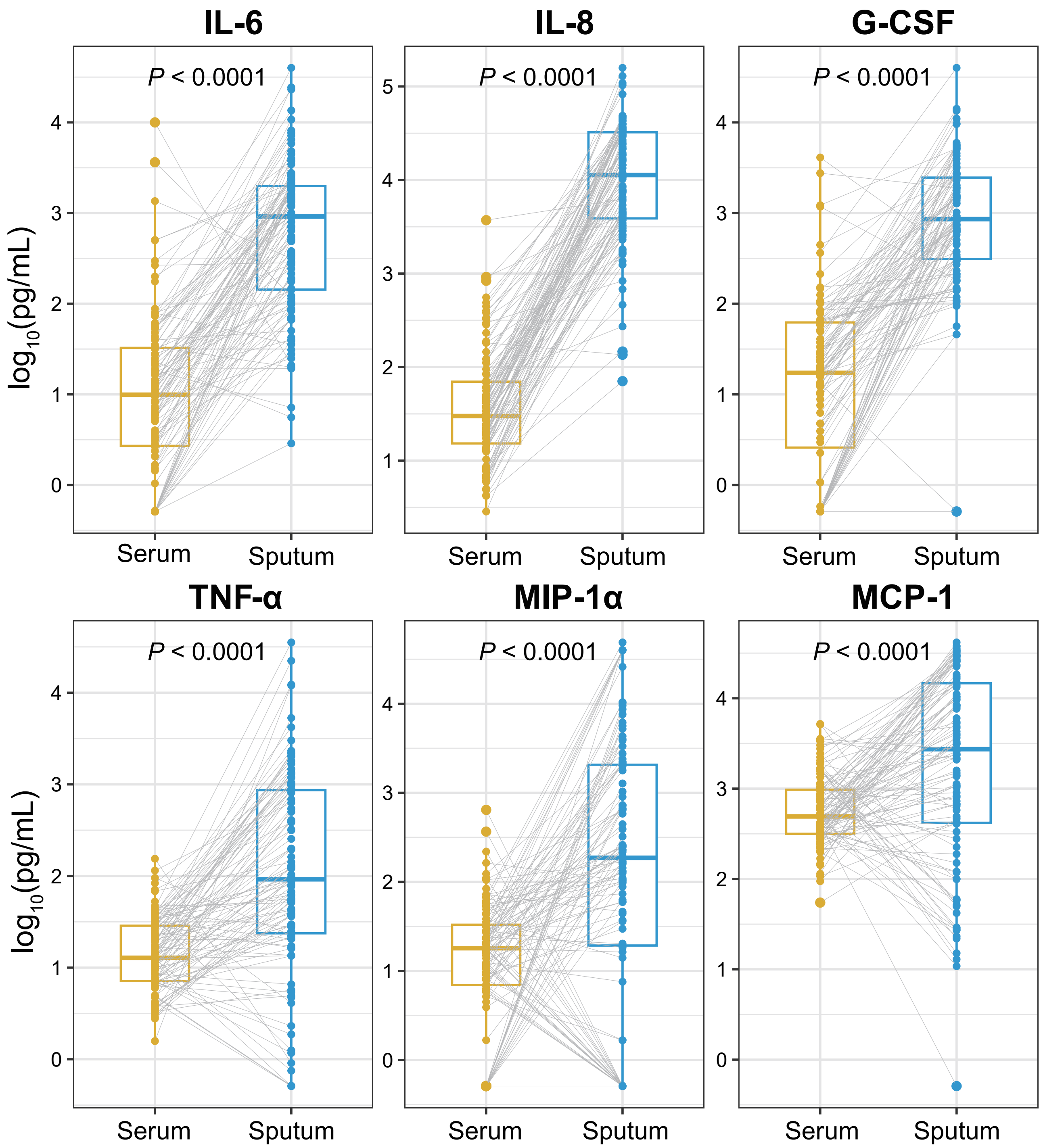

Figure S4. Comparison of cytokine concentrations in matched serum and sputum samples.

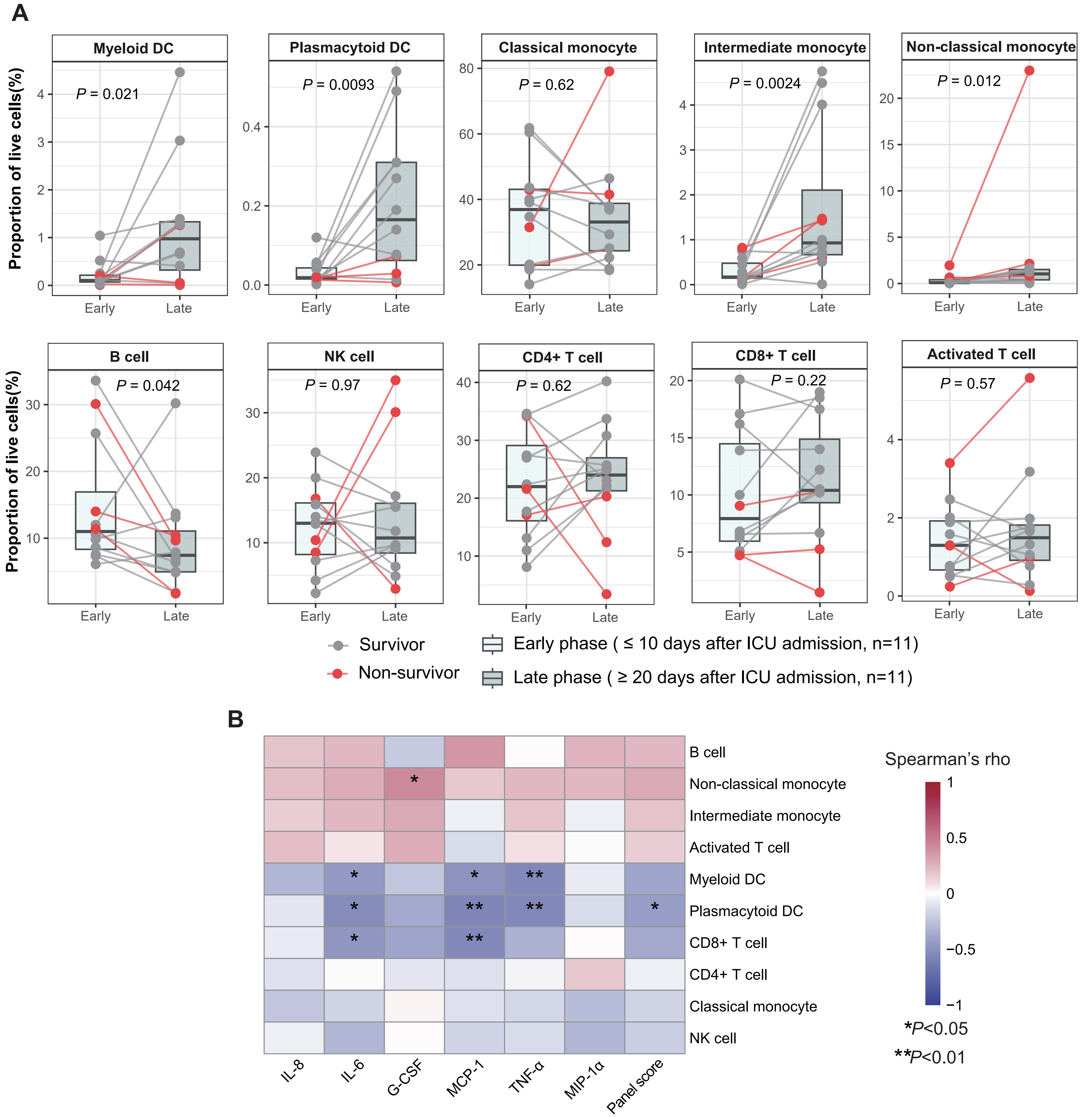

Figure S5. Longitudinal changes of circulating immune cell subsets and their correlations with the core cytokine panel.

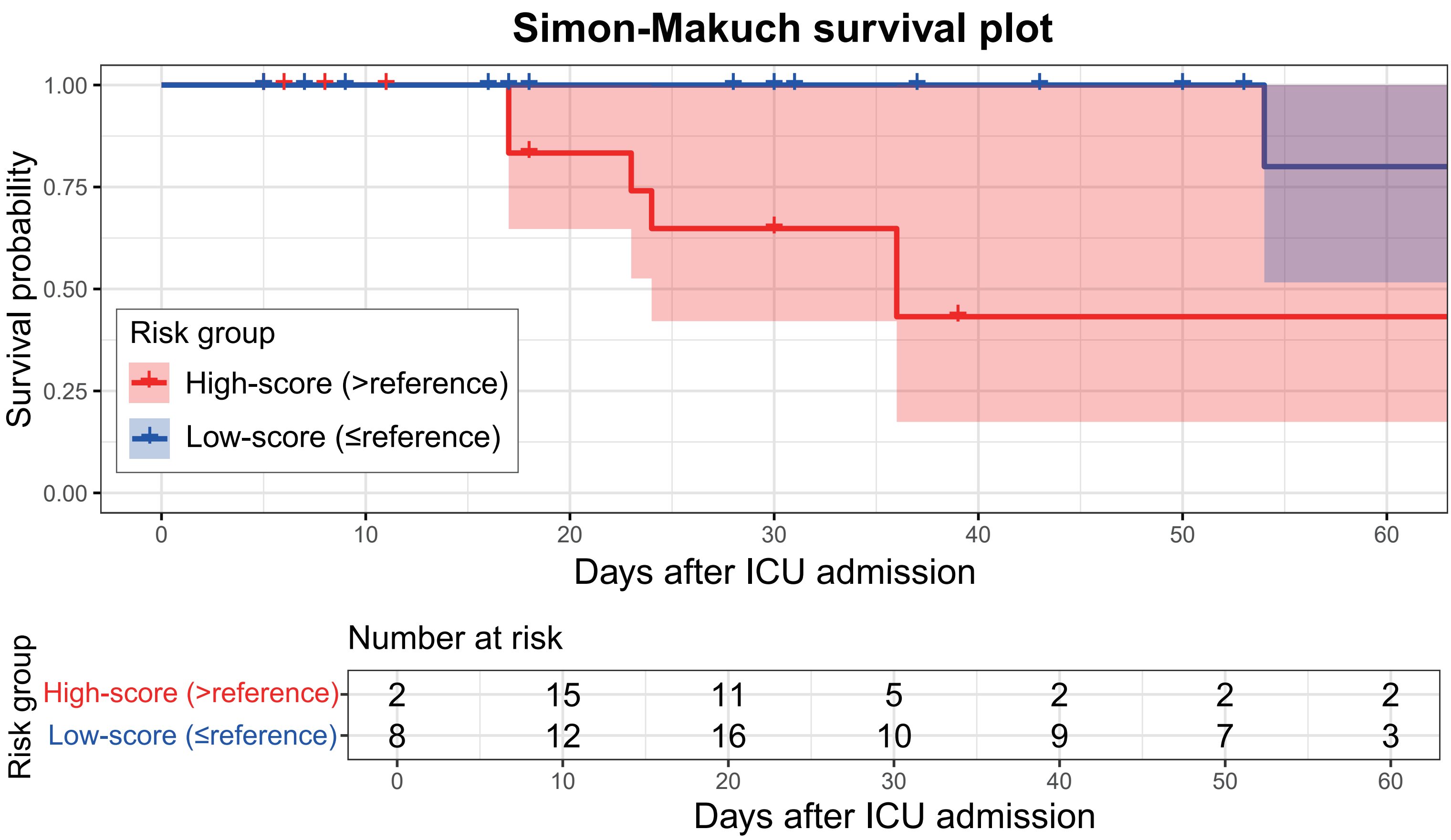

Figure S6. Simon-Makuch survival plot stratified by the time-updated core panel scores.

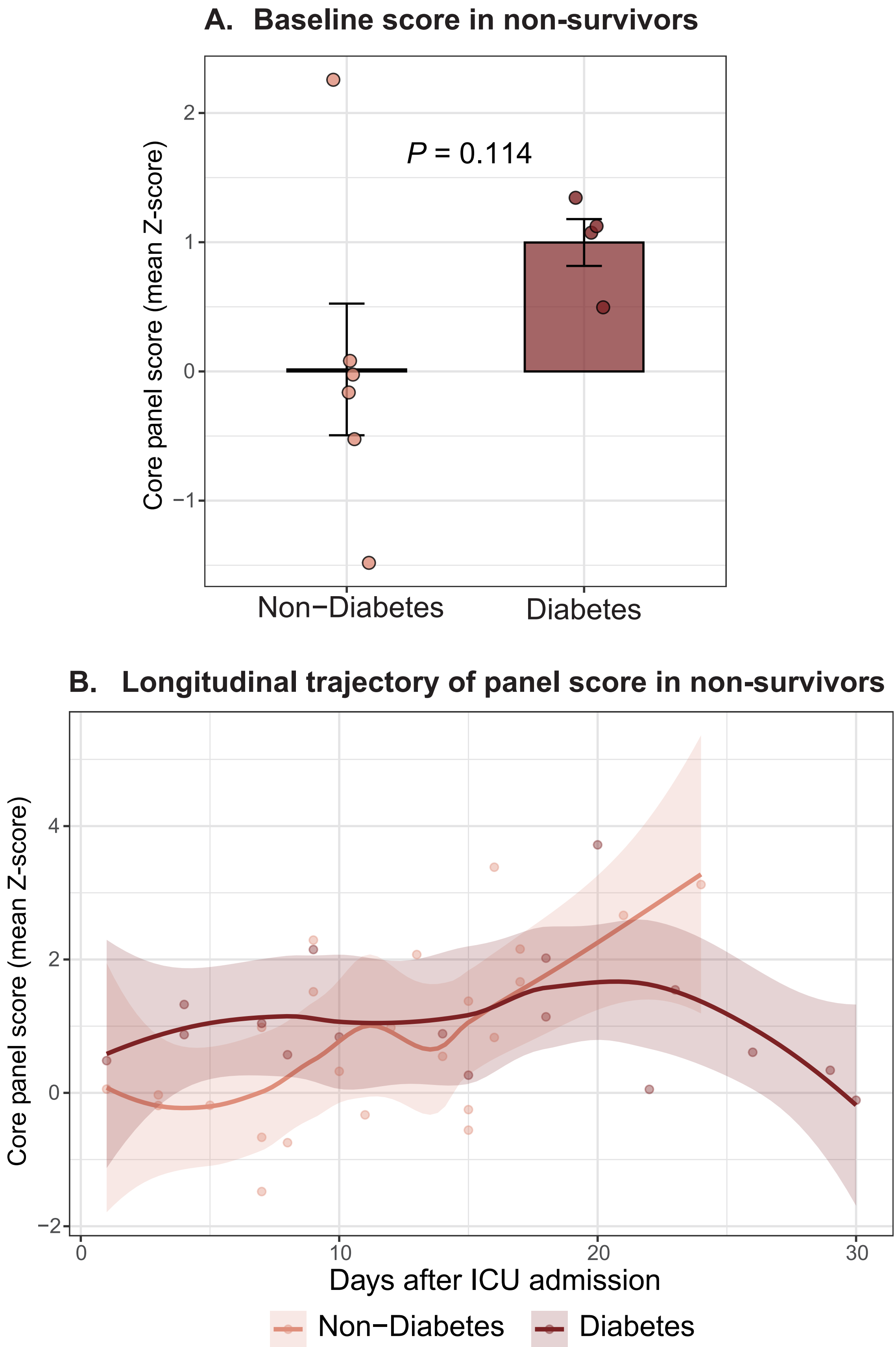

Figure S7. Cytokine panel scores in non-survivors stratified by diabetes status.

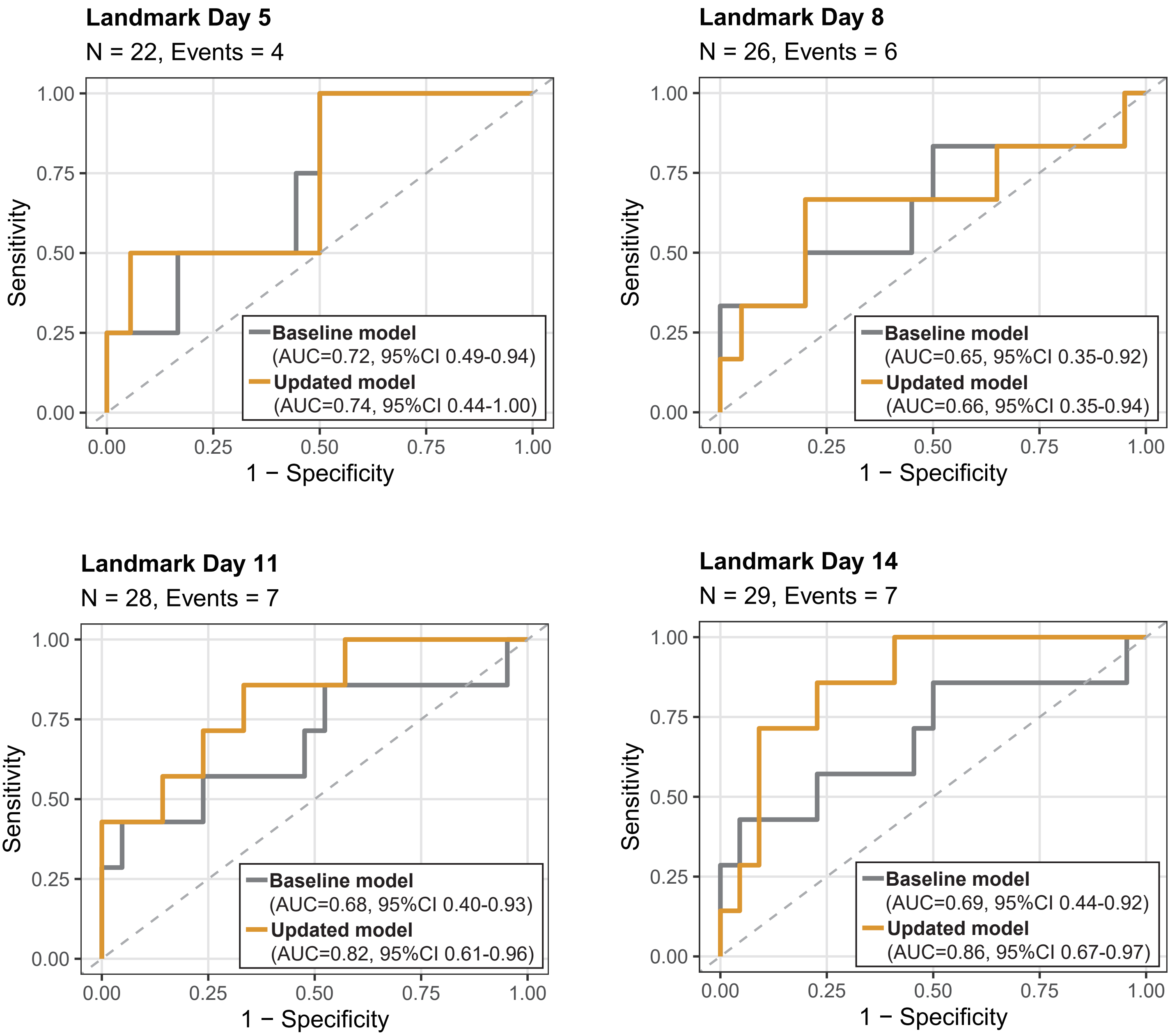

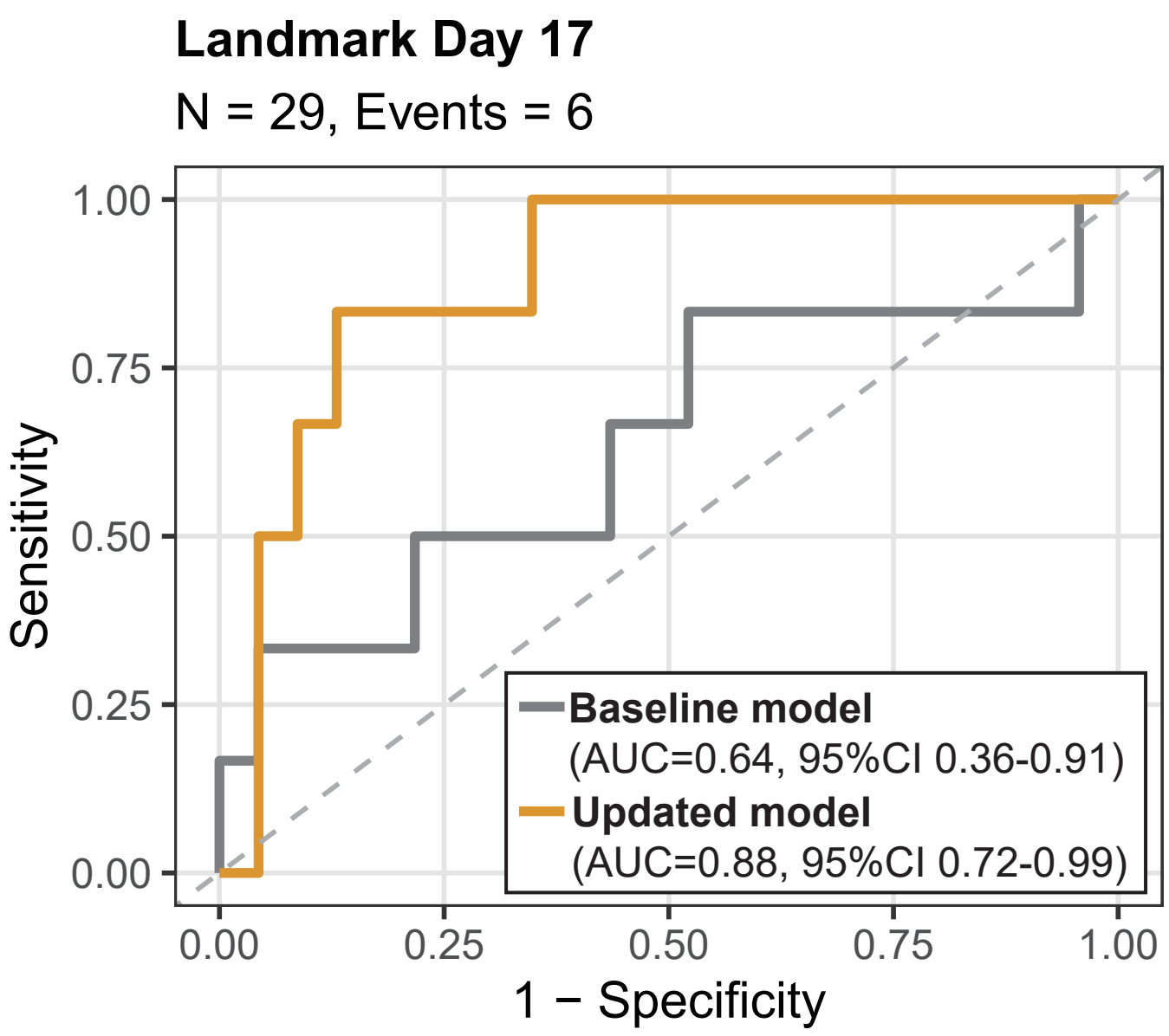

Landmark day	Baseline model		Updated model	
	Sensitivity	Specificity	Sensitivity	Specificity
5	1.00	0.50	1.00	0.50
8	0.83	0.50	0.67	0.80
11	0.43	0.95	0.86	0.67
14	0.43	0.95	0.86	0.77
17	0.83	0.48	0.83	0.87

Figure S8. Receiver operating characteristic curves and classification metrics of baseline/updated models at each landmark time.

Supplementary figure legends

Figure S1. Longitudinal trajectories of LODS scores in LSFLU cohort.

The plot displays the temporal changes in logistic organ dysfunction system (LODS) scores for survivors (blue) and non-survivors (red) over days after symptom onset. Solid lines represent the fitted trajectories, and shaded areas indicate the 95% confidence intervals.

Figure S2. Longitudinal trajectories of serum cytokines in the LSFLU cohort.

Temporal kinetics of all measured serum cytokines are shown for survivors (blue) and non-survivors (red). Cytokine concentrations were log10-transformed. Solid lines represent the fitted trajectories, and shaded areas indicate the 95% confidence intervals. Individual data points are shown as dots.

Figure S3. Sputum cytokines longitudinally associated with lung injury severity.

(A) Association between sputum cytokines and ventilator-free days. Volcano plot derived from LMM assessing the association between sputum cytokine levels and disease severity (high-VFD vs. low-VFD), adjusted for age, sampling time points, lung comorbidities, and virus type.

(B) Forest plot displaying the top 10 sputum cytokines most significantly associated with severe lung injury (defined by low ventilator-free days, VFD), ranked by P-value. The points and error bars represent the fixed effect estimates (log10 difference) and 95% confidence intervals derived from linear mixed models comparing the low-VFD group (severe injury) to the high-VFD group. All listed cytokines were significantly elevated in patients with severe lung injury.

Figure S4. Comparison of cytokine concentrations in matched serum and sputum samples.

Paired boxplots showing the levels of six core cytokines in serum and sputum collected from the same time points (28 patients, N=107 pairs). Paired comparisons were performed using the Wilcoxon signed-rank test.

Figure S5. Longitudinal changes of circulating immune cell subsets and their correlations with the core cytokine panel.

(A) Paired flow cytometric analysis of circulating immune cell subsets in 11 patients. Comparisons were performed using the Wilcoxon signed-rank test.

(B) Heatmap showing Spearman’s correlation coefficients between the core cytokine panel and circulating immune cell subsets.

Figure S6. Simon-Makuch survival plot stratified by the time-updated core panel scores.
Survival trajectories within 60 days after ICU admission were estimated using the Simon-Makuch method to account for the time-varying nature of the inflammatory panel score. Patients were dynamically classified into High-score group (red line, score > reference mean) or Low-score group (blue line, score ≤ reference mean) categories based on their most recent measurement. The shaded areas represent 95% confidence intervals. The plot is truncated at 60 days post-ICU admission. The table below displays the number of patients at risk in each state at specific time points.

Figure S7. Cytokine panel scores in non-survivors stratified by diabetes status.
(A) Comparisons of baseline panel scores between non-survivors with and without diabetes. The Mann-Whitney U test was used for statistical analysis.
(B) Longitudinal trajectories of core panel scores in non-survivors with and without diabetes. Dots represent individual core panel scores after ICU admission. Solid lines show the LOESS regression curves illustrating temporal trends in non-survivors with (dark red) or without (light red) diabetes. Shaded areas indicate 95% confidence intervals.

Figure S8. Receiver operating characteristic curves and classification metrics of baseline/updated models at each landmark time.
ROC curves were shown for the baseline model and updated model at landmark days 5, 8, 11, 14, and 17 after ICU admission in the LSFLU cohort. AUC values with 95% confidence intervals were annotated in each panel. Sensitivity and specificity values for both models were summarized within the figure for reference.
