# Supplementary material for: Dynamic monitoring of a core pro-inflammatory cytokine panel improves mortality prediction in severe influenza
Source: Front Immunol. 2026 Aug 19;17:1805650. doi: 10.3389/fimmu.2026.1805650 (PMC13533885; doi:10.3389/fimmu.2026.1805650)
Supplement: Supplementary file 2 [file DataSheet2.docx]

**Supplementary Methods: Flow cytometry**

Peripheral blood mononuclear cells (PBMC) obtained from 11 patients in the LSFLU cohorts were analyzed by flow cytometry to assess longitudinal changes in innate and adaptive immune cell populations. Paired PBMC samples collected during the early phase of disease (within 10 days after ICU admission) and the late phase (more than 20 days after ICU admission) were included.

Cryopreserved PBMCs were stained with a viability dye and fluorochrome-conjugated monoclonal antibodies according to manufacturers’ protocols. The following reagents were used: Ghost Dye Red 780 (TONBO, #13-0865-T100), CD56-BV510 (BD, #563041), HLA-DR-BV786 (BD, #564041), CD16-BV650 (BD, #563692), CD4-BB515 (BD, #564419), CD19-BUV737 (BD, #612756), CD8-PE-Cy5 (BioLegend, #344712), CD123-PE-CF595 (BD, #562391), CD3-BUV395 (BD, #564001), CD14-BV605 (BD, #564054), CD11c-PerCP-Cy5.5 (BD, #565227), CD11b-BV711 (BioLegend, #301344), CD45RA-APC (Biolegend, #304120), and CD38-PE (BD, #555460).

After staining, samples were acquired on a BD LSRFortessa X-20 flow cytometer and analyzed using FlowJo software version 10.8.2. Doublets and dead cells were excluded before downstream analysis. Immune cell subsets were defined according to the following gating strategy: Myeloid dendritic cells: HLA-DR^+^CD14^-^CD11c^+^; Plasmacytoid dendritic cells: HLA-DR^+^CD14^-^CD123^+^; Classical monocytes: HLA-DR^+^CD14^+^CD16^-^; Intermediate monocytes: HLA-DR^+^CD14^+^CD16^+^; Non-classical monocytes: HLA-DR^+^CD14_dim_CD16^+^; NK cells: CD45RA^+^CD3^-^CD56^+^; B cells: CD45RA^+^CD3^-^CD19^+^; Activated T cells: CD45RA^+^CD3^+^CD38^+^HLA-DR^+^; CD4^+^ T cells: CD45RA^+^CD3^+^CD4^+^CD8^-^; CD8^+^ T cells: CD45RA^+^CD3^+^CD8^+^CD4^-^.
