# Supplementary material for: Dynamic monitoring of a core pro-inflammatory cytokine panel improves mortality prediction in severe influenza
Source: Front Immunol. 2026 Aug 19;17:1805650. doi: 10.3389/fimmu.2026.1805650 (PMC13533885; doi:10.3389/fimmu.2026.1805650)
Supplement: Supplementary file 3 [file Table1.docx]

**Supplementary Table S1**. Cytokines significantly altered in sepsis-complicated influenza and associated with organ dysfunction

| **Cytokine** | **Change in CWS vs. SRI** | **Association with LODS** | **Functional category** | **Reported role in severe influenza or sepsis** |
| --- | --- | --- | --- | --- |
| (1) **Cytokines consistently associated with severity progression and organ dysfunction** | | | | |
| IL-6 | ↑ | ↑ | Pleiotropic pro-inflammatory cytokine | Central driver of cytokine storm, acute phase response, and multi-organ dysfunction; robust early severity/hospitalization predictor [1-4] |
| IL-8 | ↑ | ↑ | Pro-inflammatory chemokine | Primary neutrophil chemoattractant; key mediator of pulmonary neutrophil influx, ARDS, and tissue damage [1, 3, 5, 6] |
| G-CSF | ↑ | ↑ | Neutrophil colony-stimulating factor | Promotes neutrophil mobilization/activation; linked to prolonged tissue injury and poor outcome [2, 7] |
| MCP-1 | ↑ | ↑ | Pro-inflammatory chemokine | Recruit monocytes/macrophages; amplifies cytokine storm and sustains inflammation [6, 8, 9] |
| TNF-α | ↑ | ↑ | Classical pro-inflammatory cytokine | Major amplifier of systemic inflammation; drives endothelial activation, vascular leakage, and septic shock [1, 9, 10] |
| MIP-1α | ↑ | ↑ | Pro-inflammatory chemokine | Recruit monocytes, T cells, NK cells; contributes to macrophage activation and persistent lung pathology [11-13] |
| IL-10 | ↑ | ↑ | Anti-inflammatory cytokine | Reflect compensatory immunosuppression; elevated levels correlate with late-stage immune dysregulation and poor prognosis [9, 14, 15] |
| IL-1RA | ↑ | ↑ | Anti-inflammatory (IL-1 receptor antagonist) | Endogenous negative feedback to IL-1; rises in parallel with inflammation, indicates unresolved response [16, 17] |
| IL-15 | ↑ | ↑ | Lymphocyte growth factor | Enhance NK and memory T cell activity; excessive levels contribute to immunopathology in critical illness [1, 18, 19] |
| (2) **Additional cytokines increased in CWS** | | | | |
| IP-10 | ↑ | — | Pro-inflammatory chemokine | Recruit activated T cells and NK cells; associated with sustained Th1-type inflammation [6, 11, 20] |
| Fractalkine | ↑ | — | Chemokine & adhesion molecule | Promotes leukocyte adhesion/recruitment; aggravates inflammation and multi-organ injury in sepsis [17, 21, 22] |
| IL-12P40 | ↑ | — | Subunit of IL-12/IL-23 | Promotes Th1/Th17 responses; associated with hypercapnia in ARDS patients [23, 24] |
| IL-1β | ↑ | — | Classical pro-inflammatory cytokine | Key initiator of acute inflammation, fever, and NF-κB activation [25, 26] |
| IL-2 | ↑ | — | T cell growth factor | Support T cell proliferation; increased in the hyperthermic-fast-resolver sepsis subphenotypes [9, 19] |
| IL-4 | ↑ | — | Th2-type cytokine | Promote Th2 differentiation; may reflect immune imbalance or compensatory shift in sepsis [27] |
| (3) **Additional cytokines associated with higher LODS** | | | | |
| MCP-3 | — | ↑ | Pro-inflammatory chemokine | Contributes to leukocyte infiltration [28] |
| (4) **Cytokines decreased or negatively associated with severity** | | | | |
| sCD40L | ↓ | ↓ | Platelet-derived immune modulator | Marker of platelet activation; decreased due to consumption in severe coagulopathy and endothelial damage; decreased in influenza-associated encephalopathy [29] |
| IL-12P70 | ↓ | — | Th1-polarizing cytokine | Drives Th1 antiviral response; reduced in severe septic shock (reflecting impaired Th1 immunity and immunosuppression in nonsurvivors); elevated in critical influenza patients as part of early Th1 hyperactivation [18, 30] |
| IL-5 | ↓ | — | Th2-type cytokine (eosinophil growth) | Involve in eosinophil activation; decrease reflects Th2 suppression in hyperinflammatory states [31] |
| Eotaxin | ↓ | — | Chemokine (eosinophil chemoattractant) | Recruit eosinophils; positive correlation with sepsis severity [32] |
| MDC | — | ↓ | Chemokine (Th2/Treg recruiting) | Recruit regulatory/Th2 cells; reduced in fatal H7N9 cases; elevated in infection-induced sepsis (discriminated from injury) [33, 34] |
| PDGF-AB/BB | — | ↓ | Growth factor (tissue/vascular repair) | Promotes repair and fibrosis; reduction suggests impaired resolution capacity in severe sepsis [35, 36] |

**Supplementary Table S2**. Reference values of six cytokines for core panel score calculation

|  | IL-8 | IL-6 | G-CSF | MCP-1 | TNF-α | MIP-1α |
| --- | --- | --- | --- | --- | --- | --- |
| ^†^Mean (pg/mL) | 30.79 | 8.12 | 13.30 | 567.28 | 10.80 | 7.55 |
| ^†^SD (pg/mL) | 2.69 | 4.00 | 4.26 | 1.83 | 1.74 | 5.26 |

^†^Reference values were calculated using the cytokine levels within 5 days after ICU admission, from 23 patients in the LSFLU cohort.

**Supplementary Table S3**. Proportional hazards diagnostics based on Schoenfeld residuals for Cox regression models

| **Models** | **Covariate** | **Chi-square (χ^2^)** | **df** | ***P* value** |
| --- | --- | --- | --- | --- |
| **(1) Primary baseline and time-dependent Cox regression models for the core cytokine panel** | | | | |
| Baseline model | Baseline panel score | 0.024 | 1 | 0.880 |
| Dynamic model | Baseline panel score | 1.570 | 1 | 0.210 |
|  | Delta panel score | 0.600 | 1 | 0.440 |
|  | Global | 1.630 | 2 | 0.440 |
| Current model | Current panel score | 0.013 | 1 | 0.910 |
| **(2) Conventional predictor comparison** | | | | |
| Baseline LODS | Baseline LODS | 1.200 | 1 | 0.270 |
| Current LODS | Current LODS | 1.960 | 1 | 0.160 |
| Baseline CRP | Baseline CRP | 2.030 | 1 | 0.150 |
| Current LODS+Current panel score | Current LODS | 0.005 | 1 | 0.940 |
|  | Current panel score | 0.278 | 1 | 0.600 |
|  | Global | 0.286 | 2 | 0.870 |
| **(3) Diabetes-adjusted sensitivity analyses** | | | | |
| Baseline panel score+Diabetes | Baseline panel score | 0.003 | 1 | 0.959 |
|  | Diabetes | 4.100 | 1 | 0.045 |
|  | Global | 4.519 | 2 | 0.104 |
| Current panel score+Diabetes | Current panel score | <0.001 | 1 | 0.987 |
|  | Diabetes | 3.248 | 1 | 0.072 |
|  | Global | 3.438 | 2 | 0.179 |
| Current LODS+Diabetes | Current LODS | 1.630 | 1 | 0.201 |
|  | Diabetes | 5.740 | 1 | 0.017 |
|  | Global | 7.610 | 2 | 0.022 |
| Current LODS+Current panel score  +Diabetes | Current LODS | <0.001 | 1 | 0.999 |
|  | Current panel score | 0.071 | 1 | 0.790 |
|  | Diabetes | 3.180 | 1 | 0.074 |
|  | Global | 3.630 | 3 | 0.304 |

Note: Global tests were shown for multivariable models.

**Supplementary Table S4**. Interaction analysis between panel score and virus type in Cox model

| Models & Variables | Hazard ratio | 95% CI | *P* value |
| --- | --- | --- | --- |
| **Model A: Baseline score interaction**^†^ |  |  |  |
| Baseline score × virus type | 0.28 | 0.04-2.15 | 0.222 |
| **Model B: Current score interaction**^†^ |  |  |  |
| Current score × virus type | 2.00 | 0.45-8.93 | 0.366 |

^†^The interaction term tested whether the association between panel score and mortality differed by virus type.

**References**

1.	Hagau, N., et al., Clinical aspects and cytokine response in severe H1N1 influenza A virus infection. Critical Care (London, England), 2010. 14(6): p. R203.

2.	Zhang, J., et al., Interleukin-6 and granulocyte colony-stimulating factor as predictors of the prognosis of influenza-associated pneumonia. BMC Infectious Diseases, 2022. 22(1): p. 343.

3.	Shen, Z., et al., Host immunological response and factors associated with clinical outcome in patients with the novel influenza A H7N9 infection. Clinical Microbiology and Infection : the Official Publication of the European Society of Clinical Microbiology and Infectious Diseases, 2014. 20(8): p. O493-O500.

4.	Li, X., et al., Innate immune role of IL-6 in influenza a virus pathogenesis. Frontiers In Cellular and Infection Microbiology, 2025. 15: p. 1605446.

5.	Garcinuño, S., et al., Immune dysregulation is an important factor in the underlying complications in Influenza infection. ApoH, IL-8 and IL-15 as markers of prognosis. Frontiers In Immunology, 2024. 15: p. 1443096.

6.	Betakova, T., et al., Cytokines Induced During Influenza Virus Infection. Current Pharmaceutical Design, 2017. 23(18): p. 2616-2622.

7.	Kajihara, K., et al., Systemic cytokines drive conserved severity-associated myeloid responses across bacterial and viral infections. Communications Biology, 2025. 8(1): p. 1096.

8.	Zou, Y., et al., Peripheral blood cytokine expression levels and their clinical significance in children with influenza. Translational Pediatrics, 2025. 14(2): p. 286-297.

9.	Davey, R.T., et al., The association between serum biomarkers and disease outcome in influenza A(H1N1)pdm09 virus infection: results of two international observational cohort studies. PloS One, 2013. 8(2): p. e57121.

10.	Gu, Y., et al., The Mechanism behind Influenza Virus Cytokine Storm. Viruses, 2021. 13(7).

11.	Hall, M.W., et al., Innate immune function and mortality in critically ill children with influenza: a multicenter study. Critical Care Medicine, 2013. 41(1): p. 224-236.

12.	Nguyen, T.H.O., et al., Immune cellular networks underlying recovery from influenza virus infection in acute hospitalized patients. Nature Communications, 2021. 12(1): p. 2691.

13.	Ferrero, M.R., L.P. Tavares, and C.C. Garcia, The Dual Role of CCR5 in the Course of Influenza Infection: Exploring Treatment Opportunities. Frontiers In Immunology, 2022. 12: p. 826621.

14.	Alagarasu, K., et al., TNFA and IL10 Polymorphisms and IL-6 and IL-10 Levels Influence Disease Severity in Influenza A(H1N1)pdm09 Virus Infected Patients. Genes, 2021. 12(12).

15.	Hotchkiss, R.S., et al., Sepsis and septic shock. Nature Reviews. Disease Primers, 2016. 2: p. 16045.

16.	Bermejo-Martin, J.F., et al., Host adaptive immunity deficiency in severe pandemic influenza. Critical Care (London, England), 2010. 14(5): p. R167.

17.	Fu, Z., et al., A Correlation between Sequential Organ Failure Assessment Scores and Biomarkers of Inflammation and Infection in Patients with Sepsis. Current Medicinal Chemistry, 2025.

18.	Bermejo-Martin, J.F., et al., Th1 and Th17 hypercytokinemia as early host response signature in severe pandemic influenza. Critical Care (London, England), 2009. 13(6): p. R201.

19.	Bhavani, S.V., et al., Distinct immune profiles and clinical outcomes in sepsis subphenotypes based on temperature trajectories. Intensive Care Medicine, 2024. 50(12): p. 2094-2104.

20.	Chi, Y., et al., Cytokine and chemokine levels in patients infected with the novel avian influenza A (H7N9) virus in China. The Journal of Infectious Diseases, 2013. 208(12): p. 1962-1967.

21.	Hoogendijk, A.J., et al., Plasma fractalkine is a sustained marker of disease severity and outcome in sepsis patients. Critical Care (London, England), 2015. 19: p. 412.

22.	Zhang, C., et al., Alterations in CX3CL1 Levels and Its Role in Viral Pathogenesis. International Journal of Molecular Sciences, 2024. 25(8).

23.	Lim, H.X., et al., Principal role of IL-12p40 in the decreased Th1 and Th17 responses driven by dendritic cells of mice lacking IL-12 and IL-18. Cytokine, 2013. 63(2): p. 179-186.

24.	Zhao, J., et al., Longitudinal Changes in Peripheral and Alveolar Monocyte and Inflammatory Biomarkers are Distinct in Hypercapnia Patients Following Pulmonary Sepsis-Induced ARDS. Journal of Inflammation Research, 2025. 18: p. 5217-5233.

25.	Chiaretti, A., et al., IL-1 β and IL-6 upregulation in children with H1N1 influenza virus infection. Mediators of Inflammation, 2013. 2013: p. 495848.

26.	Yazdi, A.S. and K. Ghoreschi, The Interleukin-1 Family. Advances In Experimental Medicine and Biology, 2016. 941: p. 21-29.

27.	DiPiro, J.T., et al., Association of interleukin-4 plasma levels with traumatic injury and clinical course. Archives of Surgery (Chicago, Ill. : 1960), 1995. 130(11).

28.	Santiago, J., et al., Role of monocyte chemotactic protein-3 and -4 in children with virus exacerbation of asthma. The European Respiratory Journal, 2008. 32(5): p. 1243-1249.

29.	Ichiyama, T., et al., Analysis of serum soluble CD40 ligand in patients with influenza virus-associated encephalopathy. Journal of the Neurological Sciences, 2005. 239(1): p. 53-57.

30.	Wu, H.-P., et al., Serial increase of IL-12 response and human leukocyte antigen-DR expression in severe sepsis survivors. Critical Care (London, England), 2011. 15(5): p. R224.

31.	Xie, Y., et al., Specific Cytokine Profiles Predict the Severity of Influenza A Pneumonia: A Prospectively Multicenter Pilot Study. BioMed Research International, 2021. 2021: p. 9533044.

32.	Jekarl, D.W., et al., Diagnosis and Prognosis of Sepsis Based on Use of Cytokines, Chemokines, and Growth Factors. Disease Markers, 2019. 2019: p. 1089107.

33.	Cahill, L.A., et al., Multiplexed Plasma Immune Mediator Signatures Can Differentiate Sepsis From NonInfective SIRS: American Surgical Association 2020 Annual Meeting Paper. Annals of Surgery, 2020. 272(4): p. 604-610.

34.	Liu, Y., et al., Recovered and dead outcome patients caused by influenza A (H7N9) virus infection show different pro-inflammatory cytokine dynamics during disease progress and its application in real-time prognosis. bioRxiv, 2018: p. 339333.

35.	Brueckmann, M., et al., Prognostic value of platelet-derived growth factor in patients with severe sepsis. Growth Factors (Chur, Switzerland), 2007. 25(1): p. 15-24.

36.	Wang, M., et al., Platelet-derived growth factor B attenuates lethal sepsis through inhibition of inflammatory responses. International Immunopharmacology, 2019. 75: p. 105792.
